# Identifying Older Adults at Risk of Delirium Following Elective Surgery: A Systematic Review and Meta-Analysis

**DOI:** 10.1007/s11606-017-4204-x

**Published:** 2018-01-26

**Authors:** Jennifer Watt, Andrea C. Tricco, Catherine Talbot-Hamon, Ba’ Pham, Patricia Rios, Agnes Grudniewicz, Camilla Wong, Douglas Sinclair, Sharon E. Straus

**Affiliations:** 1 Department of Geriatric Medicine, University of Toronto, 27 King’s College Circle, Toronto, Ontario M5S 1A1 Canada; 2 Institute for Health Policy Management & Evaluation, University of Toronto, 4th Floor, 155 College Street, Toronto, Ontario M5T 3M6 Canada; 3 Knowledge Translation Program, Li Ka Shing Knowledge Institute, St. Michael’s Hospital, 209 Victoria Street, East Building, Room 716, Toronto, Ontario M5B 1W8 Canada; 4 Epidemiology Division, Dalla Lana School of Public Health, University of Toronto, Health Sciences Building, 155 College Street, 6th floor, Toronto, Ontario M5T 3M7 Canada; 5 Toronto Health Economics and Technology Assessment Collaborative, Faculty of Pharmacy and Institute of Health Policy Management Evaluation, University of Toronto, 144 College Street, Toronto, Ontario M5S 3M2 Canada; 6 Telfer School of Management, University of Ottawa, 55 Laurier Avenue East, Ottawa, Ontario K1N 6N5 Canada

**Keywords:** delirium, elective surgery, perioperative medicine, older adults, prognostic factors

## Abstract

**Background:**

Postoperative delirium is a common preventable complication experienced by older adults undergoing elective surgery. In this systematic review and meta-analysis, we identified prognostic factors associated with the risk of postoperative delirium among older adults undergoing elective surgery.

**Methods:**

Medline, EMBASE, CINAHL, Cochrane Central Register of Controlled Trials, and AgeLine were searched for articles published between inception and April 21, 2016. A total of 5692 titles and abstracts were screened in duplicate for possible inclusion. Studies using any method for diagnosing delirium were eligible. Two reviewers independently completed all data extraction and quality assessments using the Cochrane Risk-of-Bias Tool for randomized controlled trials (RCTs) and the Newcastle-Ottawa Scale (NOS) for cohort studies. Random effects meta-analysis models were used to derive pooled effect estimates.

**Results:**

Forty-one studies (9384 patients) reported delirium-related prognostic factors. Among our included studies, the pooled incidence of postoperative delirium was 18.4% (95% confidence interval [CI] 14.3–23.3%, number needed to follow [NNF] = 6). Geriatric syndromes were important predictors of delirium, namely history of delirium (odds ratio [OR] 6.4, 95% CI 2.2–17.9), frailty (OR 4.1, 95% CI 1.4–11.7), cognitive impairment (OR 2.7, 95% CI 1.9–3.8), impairment in activities of daily living (ADLs; OR 2.1, 95% CI 1.6–2.6), and impairment in instrumental activities of daily living (IADLs; OR 1.9, 95% CI 1.3–2.8). Potentially modifiable prognostic factors such as psychotropic medication use (OR 2.3, 95% CI 1.4–3.6) and smoking status (OR 1.8 95% CI 1.3–2.4) were also identified. Caregiver support was associated with lower odds of postoperative delirium (OR 0.69, 95% CI 0.52–0.91).

**Discussion:**

Though caution must be used in interpreting meta-analyses of non-randomized studies due to the potential influence of unmeasured confounding, we identified potentially modifiable prognostic factors including frailty and psychotropic medication use that should be targeted to optimize care.

**Electronic supplementary material:**

The online version of this article (10.1007/s11606-017-4204-x) contains supplementary material, which is available to authorized users.

## INTRODUCTION

As our population ages, there will be a greater need for elective surgeries among older adults; however, elective surgeries can be associated with significant harm.^1^^,^ 2 Delirium is one of the most common complications of surgery among older adults, and has been associated with prolonged hospitalization, death, and admission to long-term care.^2^^–^ 4 Multifactorial interventions with a focus on optimizing mobility, vision, hearing, hydration, cognition, and sleep have proven to be effective delirium prevention strategies in medical and surgical settings.^5^^–^ 7 Therefore, identifying patients at risk of delirium is important because clinicians can develop plans to mitigate this risk. Although older adults are seen in the preoperative medicine clinic for cardiovascular and respiratory risk optimization before an elective surgery, often not enough consideration is given to risk stratification for adverse outcomes that are more common in older adults, such as delirium—despite evidence to aid in its assessment and of effective interventions for its prevention.^6^^,^ 8

Understanding delirium risk factors may help clinicians, patients, and caregivers in targeting non-pharmacological and pharmacological interventions aimed at lessening its burden. The purpose of our systematic review is to identify preoperative patient characteristics of older adults undergoing elective surgery that either predispose them to or protect them from developing postoperative delirium. We also present the pooled prevalence of postoperative delirium and the risk of delirium-related adverse outcomes from these studies. This information can be used by clinicians and patients to enhance medical decision-making and by researchers to study possible interventions aimed at lowering the incidence of postoperative delirium among older adults undergoing elective surgery.

## METHODS

This study was reported in accordance with both the PRISMA statement for reporting systematic reviews and meta-analyses and the MOOSE statement for reporting meta-analysis of observational studies in epidemiology.^9^^,^ 10 This systematic review and meta-analysis has a companion report under review at the time of publication that focuses on complications of elective surgery among older adults, excluding delirium.

### Eligibility Criteria

Prospective studies (e.g., RCTs, non-randomized controlled trials, prospective cohort studies) were eligible if they included older adults undergoing elective surgery (all patients ≥60 years old and mean patient age ≥65 years) and reported prognostic factors associated with postoperative delirium or delirium-related adverse outcomes. We limited our search to prospective studies because delirium is not well-captured in patient charts or electronic databases.^11^ Studies using any method for diagnosing postoperative delirium were eligible, and all definitions of each prognostic factor were included. Studies that included patients ≥60 years old were selected to align with definitions from the United Nations and the World Health Organization.^12^^,^ 13 Studies reporting only clinical, laboratory, or imaging investigations that are not conducted as part of routine clinical practice (i.e., measuring serum interleukin levels) were excluded, as were studies disseminated in languages other than English.

### Information Sources and Search Strategy

An experienced librarian searched MEDLINE (OVID interface, 1948 to April week 3, 2016), EMBASE (OVID interface, 1980 to April week 3, 2016), CINAHL (EBSCO interface, 1994 to April 21, 2016), Cochrane Central Register of Controlled Trials (issue 4, April 2016), and AgeLine (EBSCO interface, 1968 to April 21, 2016) for potentially relevant studies. The full search strategy for MEDLINE (eSearch 1 in Supplement 1) was modified as necessary for the other databases (full searches available upon request). The electronic search was supplemented by scanning the reference lists of included studies, searching the authors’ personal files, and contacting authors of conference proceedings.

### Study Selection

Two levels of screening were completed independently by two reviewers using *Synthesi*.SR (proprietary online software developed by the Knowledge Translation Program, Toronto, Canada): (1) screening of titles and abstracts and (2) full-text screening of articles. Initially, each reviewer independently screened 10% of a random sample of citations to ensure adequate inter-rater agreement. Study authors were contacted for further information if it was unclear whether the study met inclusion criteria. Disagreements concerning article inclusion were resolved through discussion; otherwise, a third reviewer was available to make a final decision.

### Data Abstraction

Two reviewers abstracted data independently from studies retained from level 2 screening. Study characteristics (e.g., study design) and prognostic factors associated with postoperative delirium were abstracted from included studies. Definitions operationalized by study authors for individual prognostic factors were also abstracted, where appropriate (eTable 1 in Supplement 1). Conflicts regarding the abstracted data were resolved through discussion. Authors were contacted for further information when the data were not clearly reported. When multiple studies reported data from the same source, the publication with the longest duration of follow-up was considered the major publication. If the duration of follow-up was the same for two or more publications, the study published first was considered the major publication.

### Methodological Quality Assessment

Two reviewers independently completed the quality assessment for each study using the Cochrane Risk-of-Bias Tool for RCTs or the Newcastle-Ottawa Scale (NOS) for cohort studies.^14^^,^ 15 We planned to assess other study designs with the Cochrane Effective Practice and Organization Care (EPOC) Risk-of-Bias Tool.^16^

### Statistical Methods

We calculated odds ratios (ORs), mean differences (MDs), or standardized mean differences (SMDs) to quantify the relative risk of prognostic factors associated with postoperative delirium and delirium-related complications. Whenever only continuous effect measures, such as MDs (e.g., age, body mass index) were reported by study authors, these effect measures were transformed to OR estimates, if needed, to derive an overall effect estimate that combined both dichotomous and continuous study-level effect estimates.^17^ For studies that reported multiple options with which to derive the effect estimate (e.g., 2 × 2 tables, adjusted and unadjusted ORs, MDs), the order of preference for selecting the source data is described in eTable 2 in Supplement 1.

Random effects models were used to pool incidences of delirium and derive overall effect estimates with 95% CIs when two or more studies reported extractable effect estimates. The NNF was calculated as 1/pooled incidence of delirium. Information regarding data imputation methods to approximate mean and standard deviation values is found in eMethods 1 in Supplement 1. Between-study statistical heterogeneity was quantitatively assessed with the I^2^ statistic and thresholds for its interpretation were consistent with those reported in the Cochrane Handbook for Systematic Reviews of Interventions.^18^ Subgroup analyses were conducted to explore sources of substantial between-study heterogeneity based on patient age and the type of elective surgery. A prognostic factor was considered significantly associated with postoperative delirium at a two-tailed *p*-value <0.05.

Prognostic factors that were reported in at least 10 studies were assessed for publication bias by visual inspection of funnel plots for asymmetry and with the Egger’s test.^18^^,^ 19 Where there was evidence of significant publication bias (*p* < 0.05), the trim-and-fill method was used to estimate the number of missing studies and to derive the combined effect estimates, adjusting for publication bias.^20^ All statistical analyses were conducted in R, version 3.2.4, using the *metafor* and *meta* packages.^21^^,^ 22

## RESULTS

**Figure 1:**
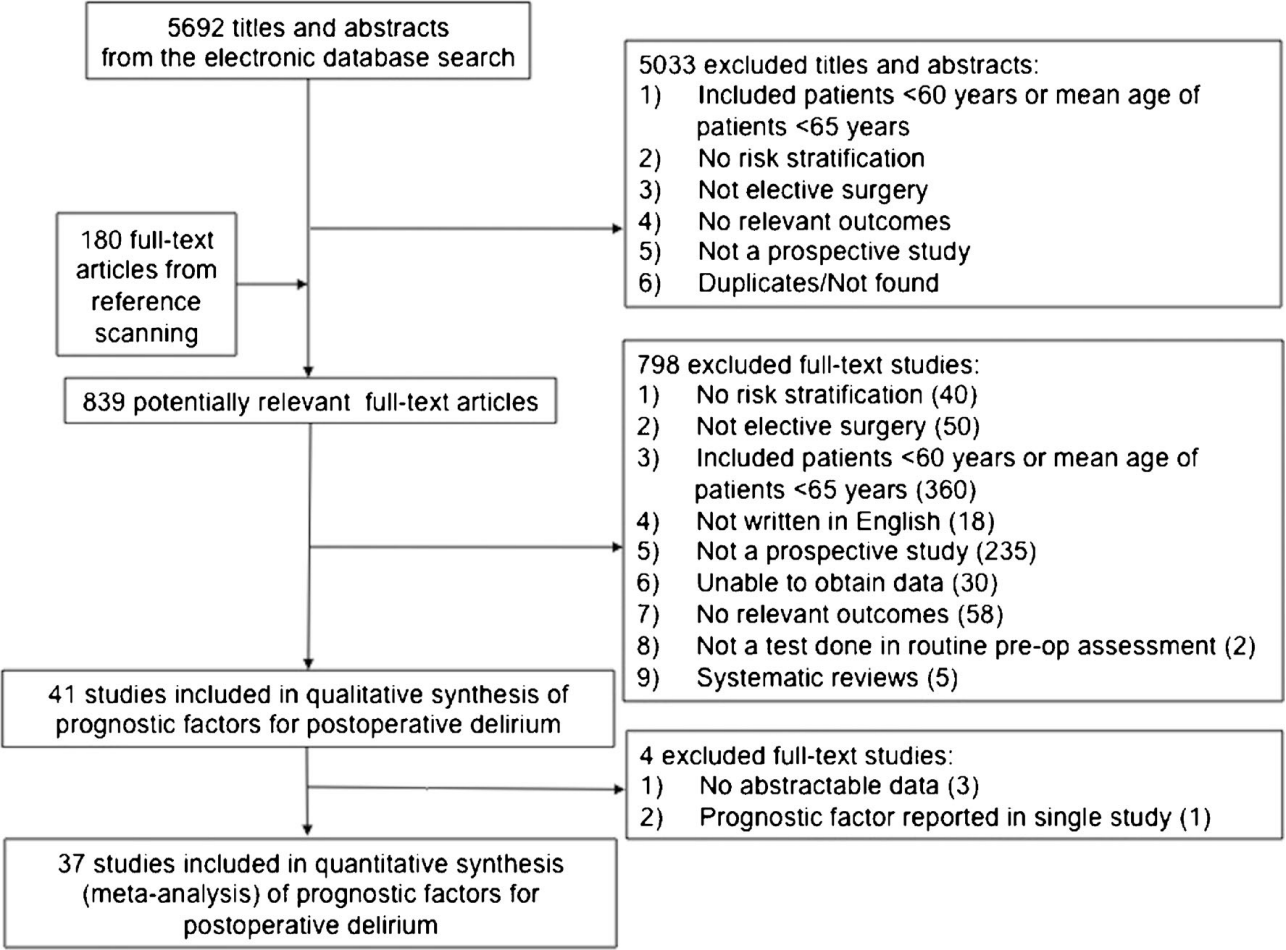
Study flow diagram outlining the number of studies 1) retrieved in our literature search, 2) excluded from our systematic review at each level of screening, and 3) included in our qualitative and quantitative synthesis of preoperative prognostic factors associated with developing postoperative delirium.

Of the 5692 titles and abstracts that were screened for inclusion, we identified 41 studies (9384 patients) that reported prognostic factors associated with the risk of developing postoperative delirium and its consequences (Fig. 1 and Table 1). Of these, 37 studies (8557 patients) were included in meta-analyses.^1^^–^ 3 ^,^ 23 ^–^ 47 ^,^ 49 ^–^ 52 ^,^ 56 ^–^ 60 In the four studies not included in our meta-analyses, data relating to prognostic factors were either not reported in an abstractable format or were reported in only one study.^61^^–^ 64 One additional study was excluded from analysis as there were data inconsistencies that precluded calculation of effect measures and we were not able to obtain further information from the study authors (see eMethods 2 in Supplement 1). One RCT was included, which was at moderate to high risk of bias (eTable 3 in Supplement 1). Overall, the included cohort studies were of moderate to high methodological quality (eTable 4 in Supplement 1). The most common biases were the adequacy of follow-up of cohorts and the failure to demonstrate that delirium was not present prior to surgery. Delirium was diagnosed by the Diagnostic and Statistical Manual of Mental Disorders (DSM) criteria (48.8%), the Confusion Assessment Method (CAM) (24.4%), both the DSM and CAM criteria (4.9%), and other methods. The method of diagnosis was not stated in 4 studies (Table 1).^65^^,^ 66

**Table 1:** Characteristics of Prospective Studies Reporting Risk Factors for Postoperative Delirium Among Older Adults Undergoing Elective Surgery

Study	Number of patients	Age range (years)	% Female	Type of surgery	Criteria for delirium	Length of follow-up (weeks)	Cases of incident delirium	
							No.	%
Rogers, 1989^23^	46	≥60	67.4	Orthopedic	Psychiatric interview for DSM-III criteria for delirium	0.57	13	28.3
Fisher, 1995^24^	80	≥60	53.8	Orthopedic	CAM	0.57	14	17.5
Rolfson, 1999^25^	71	≥65	21.3	Cardiac	CAM	0.57	23	32.4
Eriksson, 2002^26^	52	≥60	23.1	Cardiac	OBS and CAM	0.71	12	23.1
Kudoh, 2004^27^	328	65–80	81.1	Orthopedic	CAM	1	49	14.9
Santos, 2004^28^	220	61–87	35.4	Cardiac	DSM-IV criteria for delirium	0.71	74	33.6
Freter, 2005^29^	132	>65	65.2	Orthopedic	CAM	Until hospital discharge	18	13.6
Fukuse, 2005^30^	120	60–84	40	Thoracic	NR	Until hospital discharge	3	2.5
Olin, 2005^31^	51	≥65	39.2	Abdominal	CAM and medical records	Until hospital discharge	26	51
Papaioannou, 2005^32^	47	≥60	36.2	Orthopedic, urological, vascular, gynecological	DSM-III criteria for delirium	0.43	9	19.2
Rudolph, 2007^33^	1161	≥60	46.8	Orthopedic, thoracic, abdominal, vascular, other noncardiac	DSM-III criteria for delirium	1	99	8.5
Veliz-Reissmuller, 2007^2^	104	≥60	38.3	Cardiac	CAM	NR	25	23.4
Priner, 2008^34^	101	≥60	57.4	Orthopedic	CAM	NR	15	14.8
Dasgupta, 2009^35^	125	70–92	58	Orthopedic, vascular, abdominal, neurosurgical	Chart abstraction method	NR	15	12
Hattori, 2009^22^	160	>75	NR	Gastrointestinal, orthopedic, vascular	NEECHAM score < 20	1.43	87	54.7
Koebrugge, 2009^36^	71	≥65	47.9	Abdominal	DOS and DSM-IV criteria	NR	17	23.9
Morimoto, 2009^37^	20	>65	30	Abdominal	DRS ≥ 12 and DSM-IV criteria for delirium	1	5	25
Brouquet, 2010^38^	118	75–98	52.5	Abdominal	CAM	0.57	28	23.7
Kristjansson, 2010^39^	178	≥70	57	Colorectal	NR	4	14	7.9
Cerejeira, 2011^40^	101	60–89	50.5	Orthopedic	CAM and DSM-IV-TR confirmation	0.43	37	36.6
Clement, 2011^41^	1343	80–93	59.2	Orthopedic	NR	NR	17	1.3
Jankowski, 2011^42^	418	≥65	50.7	Orthopedic	CAM	12	42	10
Patti, 2011^3^	100	>65	60	Colorectal	CAM	Until hospital discharge	18	18
Tognoni, 2011^43^	90	66–93	10	Urological	CAM	1	8	8.9
Bakker, 2012^1^	201	≥70	39.8	Cardiac	CAM-ICU	1 or until hospital discharge	63	31.3
Flink, 2012^44^	106	≥65	55.7	Orthopedic	CAM and DSM-IV criteria	0.43	27	25.5
Robinson, 2012^45^	186	≥65	4.3	Abdominal, cardiac, non-cardiac thoracic, vascular	CAM-ICU	NR	102	54.8
Sasajima, 2012^46^	299	61–91	12.7	Vascular	CAM	NR	88	29.4
Gani, 2013^47^	640	≥65	3.9	Urological	CAM	NR	166	25.9
Kim, 2013^48^	141	NR	39.9	General, urological, gynecological, thoracic, breast, ophthalmologic, ENT	DSM-IV criteria	NR	14	9.9
Large, 2013^49^	49	≥65	18.4	Urological	CAM	1	14	28.6
Leung, 2013^50^	581	65–96	50.2	Orthopedic, urological, gynecological, vascular, thoracic, ENT, plastic, general	CAM	0.28	234	40.3
Otomo, 2013^51^	153	≥60	28.8	Cardiac	DRS and DSM-IV criteria for delirium	1 post-extubation	16	10.4
Kosar, 2014^52^	459	≥70	59.7	Orthopedic	CAM or validated chart-based method	Until hospital discharge	106	23.1
Suh, 2014^53^	60	70–85	100	Gynecological, general	>2/5 criteria: disorientation, inappropriate behavior, inappropriate words, delusion/hallucination, delayed psychomotor activity	4	4	6.7
Blakoe, 2015^54^	79	≥70	NR	Cardiac	NR	NR	NR	NR
Min, 2015^55^	62	65–90	29	Cardiac	New symptoms of confusion, agitation, and/or altered mental status, or new need for antipsychotic medications	NR	9	14.5
Raats, 2015^56^	83	65–89	42.2	Colorectal	DOS	NR	15	18.1
Visser, 2015^57^	463	≥60	23.1	Vascular	DOS and DSM-IV criteria	NR	22	5
Hempenius, 2016^58^	227	>65	61.9	Abdominal, gynecological, ENT, maxillofacial	DOS and DSM-IV criteria	13	26	10
Xue, 2016^59^	358	≥65	0	Urological	CAM	1	28	7.8

*Abbreviations*: CAM, Confusion Assessment Method; CAM-ICU, Confusion Assessment Method for the Intensive Care Unit; DOS, Delirium Observation Scale; DRS, Delirium Rating Scale; DSM, Diagnostic and Statistical Manual of Mental Disorders; NEECHAM, Neelon and Champagne Confusion Scale; NR, not reported; OBS, Organic Brain Syndrome Scale

### Incidence of Postoperative Delirium

The pooled incidence of postoperative delirium among included studies was 18.7% (95% CI 14.6–23.5%, 40 studies, 9305 patients, I^2^ = 95.6%, NNF = 6). The pooled incidence of delirium varied by surgical type: cardiac (23.6%, 95% CI 17–31.9%, 7 studies, I^2^ = 81.5%, NNF = 5), orthopedic (15.2%, 95% CI 8.6–25.5%, 10 studies, I^2^ = 95.3%, NNF = 7), general (26.6%, 95% CI 19.4–35.2%, 7 studies, I^2^ = 75.2%, NNF = 4), vascular (15.8%, 95% CI 7.7–29.5%, 4 studies, I^2^ = 94.2%, NNF = 7), and urological surgery (12.7%, 95% CI 1.8–53.8%, 2 studies, I^2^ = 98.6%, NNF = 8).

### Prognostic Factors

A history of delirium, frailty, and cognitive impairment were most strongly associated with developing postoperative delirium (Table 2 and eFigure 1 in Supplement 1). Other factors associated with developing postoperative delirium included psychotropic medication use, smoking status, older age, ASA status, and impairment in ADLs and IADLs. The availability of caregiver support was associated with a lower odds of postoperative delirium.

**Table 2:** Prognostic Factors for the Development of Postoperative Delirium Among Older Adults Undergoing Elective Surgery

Prognostic factor	Number of studies	Number of patients	Odds ratio (95% CI)	Heterogeneity (I^2^)
History of delirium	3	242	6.4 (2.2–17.9)	0
Frailty	2	303	4.1 (1.4–11.7)	0
Cognitive impairment	22	3982	2.7 (1.9–3.8)	72.9
Renal insufficiency	5	1336	2.3 (1.1–4.8)	70.9
Psychotropic medication use	6	1041	2.3 (1.4–3.6)	0
Psychiatric history	5	1512	2.2 (1.5–3.1)	0
Older age	27	6200	2.2 (1.6–3.2)	94.8
ADL impairment	9	1881	2.1 (1.6–2.6)	0
IADL impairment	2	699	1.9 (1.3–2.8)	5.2
Cerebrovascular disease	7	2205	1.8 (1.2–2.7)	0
Smoking status	9	2467	1.8 (1.3–2.4)	0
ASA status	10	2482	1.7 (1.2–2.4)	29.3
Low education	8	1973	1.5 (1.1–2.0)	19.6
Diabetes mellitus	11	2924	1.4 (1.0–2.0)	36.6
Neurological disease	3	981	1.4 (1.0–1.9)	0
Charlson comorbidity index	7	1588	0.40 (0.07–0.7)*	82.5
Caregiver support	6	1378	0.69 (0.52–0.9)	0
Male sex	25	5430	1.1 (0.76–1.4)	62.6
Alcohol consumption	12	3198	0.84 (0.57–1.2)	45.1
Dyslipidemia	5	784	0.71 (0.50–1.0)	0
Hypertension	9	2759	1.3 (0.86–1.9)	48.9
Coronary artery disease	6	1770	1.2 (0.78–1.8)	17.0
Myocardial infarction	5	1739	1.2 (0.70–1.9)	56.7
Obstructive lung disease	3	790	0.84 (0.44–1.6)	13.2
Hypoalbuminemia	2	218	4.0 (1–14.3)	73.6
Depression scale score	9	1846	1.4 (0.99–1.8)	25.6
Greater number of drugs	5	484	1.4 (0.95–2.1)	0
Heart failure	4	1504	1.4 (0.90–2.1)	0
Body mass index (BMI)	10	1869	0.10 (−0.45, 0.6)^†^	36.1
General anesthesia	10	2568	1.1 (0.80–1.4)	19.6
Pre-hospitalization	3	390	1.3 (0.77–2.2)	0

*Abbreviations*: ADL, activities of daily living; ASA, American Society of Anesthesiologists; CI, confidence interval; IADL, instrumental activities of daily living

*Reported as a standardized mean difference (SMD)

†Reported as a mean difference (MD)

### Subgroup Analyses of Prognostic Factors

The odds of developing postoperative delirium among patients aged ≥80 years were significantly increased (Table 3).^25^^,^ 32 ^,^ 36 ^,^ 43 ^–^ 45 ^,^ 49 ^,^ 58 Age was a significant prognostic factor among patients undergoing non-cardiac surgery^3^^,^ 24 ^–^ 29 ^,^ 37 ^,^ 38 ^,^ 41 ^–^ 45 ^,^ 49 ^–^ 51 ^,^ 56 ^,^ 58 ^,^ 59; however, it was not a significant prognostic factor among patients undergoing cardiac surgery.^1^^,^ 2 ^,^ 31 ^,^ 33 ^,^ 34 ^,^ 36 Because of the substantial heterogeneity among the studies conducted in the non-cardiac surgery population, subgroup analyses were conducted based on the type of surgery. A statistically significant association between age and the odds of developing postoperative delirium remained for each of the following surgical populations: orthopedic surgery, vascular surgery, urological surgery, and general surgery.

**Table 3:** Subgroup Analyses Exploring the Influence of Effect Modifiers on the Association Between Prognostic Factors and Postoperative Delirium

Subgroup	Number of studies	Number of patients	Odds ratio (95% CI)	Heterogeneity (I^2^)
a) Older age				
Aged ≥80 years	8	3161	2.3 (1.2–4.3)	56.1
Cardiac surgery	6	801	1.3 (0.96–1.96)	44.1
Non-cardiac surgery	21	5399	2.5 (1.4–3.8)	93.6
Orthopedic surgery	7	1829	1.3 (1.1–1.5)	0
Vascular surgery	2	762	5.7 (2.9–11.2)	0
Urological surgery	4	1078	2.8 (1.4–5.6)	51.9
General surgery	5	392	7.5 (1.5–37.8)	94.9
b) Cognitive impairment				
Cardiac surgery	4	529	2.3 (1.3–4.0)	0
Non-cardiac surgery	17	3267	2.5 (1.7–3.8)	75
Orthopedic surgery	6	938	2.4 (1.1–5.0)	72.2
Vascular surgery	2	762	6.4 (1.6–26.5)	66.1
Urological surgery	3	497	2.6 (1.6–4.2)	13.6
General surgery	3	209	1.2 (0.45–3.0)	68

Cognitive impairment was a significant risk factor for postoperative delirium in patients undergoing cardiac or non-cardiac surgery.^24^^–^ 27 ^,^ 29 ^,^ 32 ^,^ 37 ^,^ 38 ^,^ 41 ^–^ 44 ^,^ 50 ^–^ 52 ^,^ 56 ^,^ 59 Cognitive impairment was also a risk factor for delirium in patients undergoing orthopedic surgery, vascular surgery, and urological surgery; however, cognitive impairment among patients undergoing general surgery was not associated with development of postoperative delirium.

In a subgroup analysis of patients who reported a history of alcohol abuse, there was no significant effect on the development of postoperative delirium (OR 1.00, 95% CI 0.56–1.8, 4 studies, I^2^ = 0%); however, among a subgroup of current smokers, there was an association between smoking and postoperative delirium (OR 2.8, 95% CI 1.5–5.3, 3 studies, I^2^ = 0%).

### Sensitivity Analyses

Sensitivity analyses were conducted that included studies reporting ORs adjusted for potentially important confounders. Among studies reporting a significant association in the original meta-analyses, only renal insufficiency was no longer associated with increased odds of postoperative delirium (OR 2.0, 95% CI 0.44–9.6, 2 studies, I^2^ = 87.3%); however, the two studies included in this summary effect measure were clinically heterogeneous. The study by Bakker et al. reported renal insufficiency as a measure of serum creatinine level and found no significant association between renal insufficiency and postoperative delirium, whereas the study by Sasajima et al., reported the number of patients with end-stage renal disease, which was significantly associated with postoperative delirium.^1^^,^ 59 Following sensitivity analysis, a significant association remained for both age (OR 2.0, 95% CI 1.3–3.3, 9 studies, I^2^ = 96.1%) and cognitive impairment (OR 2.9, 95% CI 1.6–5.3, 10 studies, I^2^ = 78.9%). Additionally, when only those studies reporting the age-adjusted odds of postoperative delirium among older adults with cognitive impairment were included in the sensitivity analysis, there was still a significant association (OR 3.5,95% CI 1.5–7.9, 8 studies, I^2^ = 86.5%). Among the prognostic factors that were not significantly associated with postoperative delirium in the original meta-analyses, only the association between hypertension and postoperative delirium was statistically significant (OR 3.1, 95% CI 1.4–7.0, 2 studies, I^2^ = 6.1%). There were a number of studies that reported only unadjusted ORs, which precluded sensitivity analysis (eTable 5 in Supplement 1).

### Assessment for Publication Bias

There was evidence suggesting small study bias among those studies reporting cognitive impairment (*p* = 0.02). It was estimated that five studies with non-significant results may have been suppressed, although even after adjusting for these potentially missing studies, cognitive impairment remained a significant risk factor (OR 2.2, 95% CI 1.5–3.2, I^2^ = 76.3%, *p* < 0.001; eFigure 2 in Supplement 1).

### Delirium-Related Adverse Outcomes

Among studies reporting preoperative prognostic factors associated with developing postoperative delirium, postoperative delirium was significantly associated with postoperative mortality, postoperative complications, length of hospitalization, and discharge to an institution or care facility. The association between postoperative delirium and subsequent hospital readmission was reported in two studies (Table 4 and eFigure 1 in Supplement 1).^26^^,^ 44

**Table 4:** Risk of Adverse Outcomes in Patients Who Developed Postoperative Delirium

Outcome	Number of studies	Number of patients	Odds ratio (95% CI)	Heterogeneity (I^2^)
Postoperative mortality	8	951	4.0 (2.0–8.1)	14.7
Postoperative complications	7	929	3.1 (1.6–5.7)	35.0
Non-home discharge	2	656	8.7 (3.9–19.4)	27.7
Length of hospitalization	7	743	2.8 (0.7–4.9)*	73.7
Hospital readmission	2	276	2.2 (0.1–44.6)	88.7

*Abbreviations*: CI, confidence interval

*Reported as a mean difference

## DISCUSSION

Our systematic review demonstrated that postoperative delirium occurs frequently in the setting of elective surgery among older adults (NNF = 6). We also identified prognostic factors associated with the risk of postoperative delirium in this patient population. Common geriatric syndromes, including cognitive and functional impairment, were shown to be important prognostic factors that are identifiable during the preoperative assessment of older adults. Findings from our systematic review support the targeting of potentially modifiable (e.g. smoking, frailty) and protective (caregiver support) factors to optimize care. Furthermore, we showed that delirium is associated with adverse outcomes that can severely impact both the quality and quantity of life experienced by older adults following elective surgery.

In addition to non-pharmacological delirium prevention strategies that have demonstrated effectiveness in both the medical and surgical settings, other potential targets for intervention were identified in this study that warrant further research.^6^^,^ 7 For example, smoking status, frailty, and the use of psychotropic medications are potentially modifiable prognostic factors that were associated with developing postoperative delirium. Interventions for preoperative smoking cessation have been associated with a lower risk of postoperative complications.^67^ Direct patient education has also been effective at reducing benzodiazepine prescriptions.^68^

Although some would argue that there are no effective interventions for the treatment of frailty, researchers have shown that the progression of frailty is potentially preventable.^69^^,^ 70 Indeed, multicomponent interventions aimed at improving nutrition, physical fitness, and cognition have shown promise in reversing frailty.^71^ Even exercise-based interventions alone have been shown to improve frailty status.^72^^,^ 73 The application of an exercise-based intervention in the preoperative setting, termed “prehabilitation”, is currently being studied to determine whether frail patients in the perioperative setting can derive similar benefit.^74^ If such an intervention proves successful, frailty could be identified in the preoperative clinic in order to offer patients prehabilitation in anticipation of their elective surgery.

Importantly, the availability of a caregiver in the perioperative period was shown to be protective against developing postoperative delirium. This is consistent with a randomized trial in older adults with hip fractures, which showed that a non-pharmacological multicomponent intervention provided by caregivers in the perioperative setting was associated with a decreased incidence of delirium.^75^ Previous work on the subjective experience and satisfaction of patients during the perioperative period has also highlighted the provision of information and education as very important to improving patient care.^54^ This information should be communicated to patients and their caregivers as part of their preoperative clinic visit, and this finding should potentially be added to clinical practice guidelines concerning the perioperative care of older adults.^48^

In contrast to recent systematic reviews and meta-analyses on adverse postoperative outcomes among older adults, which have included patients with various indications for surgery such as hip fracture or other emergency procedures, we targeted older adults undergoing elective surgery because of the potential to intervene in the preoperative setting to improve patient outcomes.^53^^,^ 55 Contrary to our finding that smoking status was associated with developing postoperative delirium, Scholz et al. found no such association among patients aged 50 years and older undergoing any elective or emergency gastrointestinal surgery; however, their result was based on just two studies and 218 patients, while our meta-analysis included nine studies and 2467 patients.^53^ There are several potential explanations for the association between smoking status and postoperative delirium, including nicotine withdrawal during hospitalization, or perhaps these patients have a higher burden of cardiometabolic disease with as yet undiagnosed vascular cognitive impairment. For example, greater agitation was reported in a study of intensive care unit patients experiencing nicotine withdrawal; however, the association between smoking and delirium remains unclear in the broader body of literature outside the perioperative setting.^76^^,^ 77 Similar to our current study, a meta-analysis by Witlox et al. found that patients who developed postoperative delirium experienced increased postoperative mortality and institutionalization.^4^

There were limitations in our study’s review process. First, only studies that were published in English were included in this review to increase feasibility, but our findings are likely generalizable given the number of geographical regions represented in our systematic review. Second, there was substantial heterogeneity among studies for some outcomes, which could not always be adequately explored given a limited number of studies and a lack of individual patient-level data. There were also limitations in the studies themselves. The methodological quality assessment demonstrated that a number of studies reported varying lengths and intensity of follow-up, which may have impacted the incidence of delirium reported. Although some studies reported only unadjusted effect measures, which limited their ability to account for possible confounders, our sensitivity analyses demonstrated that our findings were largely consistent when only study-level effect estimates adjusted for important confounders were included in the meta-analyses.

Our study had a number of strengths. Forty-one studies and over 9000 patients were included in our systematic review and meta-analysis, which allowed us to investigate a number of possible protective and predisposing factors. The hypothesis-generating nature of this study allowed for the identification of important prognostic factors, including the availability of caregiver support, which has potentially important clinical and policy implications for patient care.

In summary, postoperative delirium is a common (NNF = 6), yet preventable, complication experienced by older adults undergoing elective surgery that can lead to prolonged hospitalization, the inability to return home, and death. This systematic review and meta-analysis identified protective and modifiable prognostic factors, including smoking, frailty, and psychotropic medication use, which should be further studied to develop interventions aimed at mitigating potential harm.

## Electronic supplementary material

ESM 1 (DOCX 1580 kb)

ESM 2 (DOCX 28 kb)
