# Supplementary material for: Identifying Older Adults at Risk of Delirium Following Elective Surgery: A Systematic Review and Meta-Analysis
Source: J Gen Intern Med. 2018 Jan 26;33(4):500–9. doi: 10.1007/s11606-017-4204-x (PMC5880753; doi:10.1007/s11606-017-4204-x)
Supplement: Supplementary file 1 — (DOCX 1580 kb) [file 11606_2017_4204_MOESM1_ESM.docx]

Supplementary Online Content

[eSearch 1. MEDLINE search strategy 2](#_Toc492987718)

[eTable 1. Definitions of prognostic factors associated with postoperative delirium among older adults undergoing elective surgery 3](#_Toc492987719)

[eTable 2. Order preference for combining data types in meta-analyses 5](#_Toc492987720)

[eTable 3. Cochrane risk of bias assessment for randomized trials 6](#_Toc492987721)

[eTable 4. Newcastle-Ottawa scale for evaluating the quality of cohort studies 7](#_Toc492987722)

[eTable 5. Table of sensitivity analyses of prognostic factors associated with postoperative delirium among older adults undergoing elective surgery 10](#_Toc492987723)

[eMethods 1. Data imputation methods 11](#_Toc492987724)

[eMethods 2. Excluded study - explanation and data imputation methods trialed with examples 12](#_Toc492987725)

[eFigure 1. Forest plots of study-level and overall effect estimates for prognostic factors associated with postoperative delirium among older adults undergoing elective surgery 16](#_Toc492987726)

[eFigure 2. Funnel plots of prospective studies reporting prognostic factors associated with postoperative delirium among older adults undergoing elective surgery 79](#_Toc492987727)

[References 89](#_Toc492987728)

# eSearch 1. MEDLINE search strategy

Database: Ovid MEDLINE(R) <1948 to April Week 23 2016>, Ovid MEDLINE(R) In-Process & Other Non-Indexed Citations <April 21, 2016> Search Strategy:
--------------------------------------------------------------------------------
1 (perioperative or peri-operative).mp.
2 (preoperative or pre-operative).mp.
3 (preadmission or pre-admission).mp.
4 or/1-3
5 (geriatrics or geriatric).mp.
6 (elderly or senior? or (old adj age) or (older adj adult?)).mp.
7 Health Services for the Aged/
8 or/5-7
9 Geriatric Assessment/
10 assessment?.mp.
11 or/9-10
12 4 and 8 and 11
13 (animals not (humans and animals)).sh.
14 12 not 13

# eTable 1. Definitions of prognostic factors associated with postoperative delirium among older adults undergoing elective surgery

| **Prognostic Factor** | **Definitions (As Per Individual Study Authors)** |
| --- | --- |
| History of Delirium | Any prior diagnosis of delirium (e.g. perioperative, in-hospital) |
| Frailty | Higher frailty on an accepted frailty assessment (e.g. Edmonton Frailty Scale) |
| Cognitive Impairment | Hx of dementia, lower cognitive test score (e.g. MMSE <24) |
| Renal Insufficiency | Hx of chronic kidney disease, serum level of serum creatinine |
| Psychotropic Medication Use | Current use of any psychoactive medications (e.g. anxiolytic/hypnotics, antipsychotics) |
| Psychiatric Hx | Hx of any psychiatric disease (excluding delirium) |
| Older Age | Higher age (e.g. dichotomized at an age cut-point, average age of patients with delirium vs. those without delirium) |
| ADL Impairment | Higher ADL dependency scale score (e.g. Barthel index) |
| IADL Impairment | Higher IADL dependency scale score (e.g. Lawton IADL scale) |
| Cerebrovascular Disease | Hx of stroke, evidence of cerebrovascular disease on cranial imaging |
| Smoking Status | Current or previous smoker, number of pack-years |
| ASA Status | Higher ASA score |
| Low Education | Lower educational attainment (e.g. high school vs. post-secondary education) |
| Diabetes Mellitus | Hx of diabetes mellitus |
| Neurological Disease | Hx of any neurological disease |
| Charlson Comorbidity Index | Higher Charlson Comorbidity Index score |
| Caregiver Support | Having a spouse, more frequent visitation by caregivers |
| Male Sex | Identified as being of the male sex on clinical assessment |
| Alcohol Consumption | Hx or current alcohol consumption at or above recommended daily limits (per patient's sex) |
| Dyslipidemia | Hx of dyslipidemia |
| Hypertension | Hx of hypertension |
| Coronary Artery Disease | Hx of any coronary artery disease |
| Myocardial Infarction | Hx of myocardial infarction |
| Obstructive Lung Disease | Hx of obstructive lung disease (e.g. chronic obstructive lung disease) |
| Hypoalbuminemia | Lower serum level of albumin, serum albumin level <30g/L |
| Depression Scale Score | Higher depression scale score (e.g. Geriatric Depression Scale) |
| Greater Number of Drugs | Higher number of medications |
| Heart Failure | Hx of heart failure |
| Body Mass Index (BMI) | Higher BMI |
| General Anesthesia | Planned use of general anesthesia during the operative procedure |
| Prehospitalization | Planned admission to hospital prior to elective surgery |
| **Abbreviations:** ADL – Activities of daily living; ASA – American Society of Anesthesiologists; CGA - Comprehensive Geriatric Assessment; CI – Confidence interval; Hx – history; IADL – Instrumental activities of daily living; MMSE - Mini Mental State Exam | |

# eTable 2. Order preference for combining data types in meta-analyses

| **Type of Data** | **Example of Data Types** | **Pooling Preference Across Dichotomized Data** | **Pooling Preference Across All Data Types** |
| --- | --- | --- | --- |
| Raw Data | Sex, Presence of Absence of a Medical Disease | 2 | 3 |
| Unadjusted Odds Ratio | Sex, Presence of Absence of a Medical Disease | 3 | 4 |
| Adjusted Odds Ratio | Categorical or Continuous Data as Reported in a Multivariable Regression Model | 1 | 1 |
| Mean Difference | Age, Body Mass Index | Not applicable | 2 |
| Standardized Mean Difference | Combining Different Scales for Cognitive Impairment | Not applicable | 2 |

**Note:** Data reported as a mean difference or standardized mean difference was transformed into an odds ratio using the method of Sanchez-Meca et al., 2003, in the metafor package.^1^

# eTable 3. Cochrane risk of bias assessment for randomized trials

| **Author, Year** | **Random sequence generation** | **Allocation concealment** | **Blinding of participants and personnel** | **Blinding of outcome assessment** | **Incomplete outcome data** | **Selective reporting** | **Other bias** |
| --- | --- | --- | --- | --- | --- | --- | --- |
| Papaioannou, 2005^2^ | Low Risk | Unclear Risk | Unclear Risk | Unclear Risk | Low Risk | Low Risk | High Risk |

# eTable 4. Newcastle-Ottawa scale for evaluating the quality of cohort studies

| **Author, Year** | **Representative exposed cohort** | **Selection of the non- exposed cohort** | **Ascertainment of exposure** | **Demonstration that outcome of interest was not present at start of study** | **Comparability of cohorts on the basis of the design or analysis** | **Assessment of outcome** | **Follow-up long enough for outcomes to occur** | **Adequacy of follow up of cohorts** |
| --- | --- | --- | --- | --- | --- | --- | --- | --- |
| Bakker, 2012^3^ | * | * | * | * | ** | * | * | * |
| Blakoe, 2015^4^ | * | * | * |  |  | * |  | * |
| Brouquet, 2010^5^ | * | * | * |  | * | * | * |  |
| Cerejeira, 2011^6^ | * | * | * | * | * | * |  | * |
| Clement, 2011^7^ | * | * | * |  |  |  |  |  |
| Dasgupta, 2009^8^ | * | * | * |  | * | * |  |  |
| Eriksson, 2002^9^ | * | * | * | * |  | * | * | * |
| Fisher, 1995^10^ | * | * | * |  | * | * | * | * |
| Flink, 2012^11^ | * | * | * | * | * | * |  | * |
| Freter, 2005^12^ | * | * | * |  | ** | * | * |  |
| Fukuse, 2005^13^ | * | * | * |  | * |  | * |  |
| Gani, 2013^14^ | * | * | * |  |  | * |  |  |
| Hattori, 2009^15^ | * | * | * | * | ** | * | * |  |
| Hempenius, 2016^16^ | * | * | * |  |  |  | * | * |
| Jankowski, 2011^17^ | * | * | * | * | ** | * | * | * |
| Kim, 2013^18^ | * | * | * |  | * | * |  |  |
| Koebrugge, 2009^19^ | * | * | * | * | ** | * | * | * |
| Kosar, 2014^20^ | * | * | * | * | ** | * | * | * |
| Kristjansson, 2010^21^ | * | * | * |  |  | * | * | * |
| Kudoh, 2004^22^ | * | * | * |  |  | * | * |  |
| Large, 2013^23^ | * | * | * | * | ** | * | * | * |
| Leung, 2013^24^ | * | * | * |  | ** | * |  | * |
| Min, 2015^25^ | * | * | * | * | * | * | * | * |
| Morimoto, 2009^26^ | * | * | * |  |  | * | * | * |
| Olin, 2005^27^ | * | * | * | * |  | * | * | * |
| Otomo, 2013^28^ | * | * | * |  | * | * | * |  |
| Patti, 2011^29^ | * | * | * | * | ** | * | * | * |
| Priner, 2008^30^ | * | * | * |  | ** | * | * |  |
| Raats, 2015^31^ | * | * | * |  |  | * | * |  |
| Robinson, 2012^32^ | * | * | * |  |  | * |  |  |
| Rogers, 1989^33^ | * | * | * |  | ** | * | * | * |
| Rolfson, 1999^34^ | * | * | * |  | * | * | * | * |
| Rudolph, 2007^35^ | * | * | * |  | ** | * | * |  |
| Santos, 2004^36^ | * | * | * |  | ** | * | * |  |
| Sasajima, 2012^37^ | * | * | * |  | ** | * |  | * |
| Suh, 2014^38^ | * | * | * |  | ** | * | * | * |
| Tai, 2015^39^a | * | * | * |  |  | * | * |  |
| Tognoni, 2011^40^ | * | * | * |  |  | * | * |  |
| Veliz-Reissmuller, 2007^41^ | * | * | * | * | ** | * | * | * |
| Visser, 2015^42^ | * | * | * |  | ** | * | * |  |
| Xue, 2016^43^ | * | * | * | * | ** | * | * | * |
| a This study was excluded from analysis due to concerns over the accuracy of the reported data | | | | | | | | |

**Newcastle-Ottawa scale for evaluating the quality of cohort studies**

*Note: A study can be awarded a maximum of one star for each numbered item within the Selection and Outcome categories. A maximum of two stars can be given for Comparability.*

**Selection**

1. Representativeness of the exposed cohort

a) truly representative of the average older adult in the community *****

b) somewhat representative of the average older adult in the community *****

c) selected group of users (e.g., nurses, volunteers)

d) no description of the derivation of the cohort

2. Selection of the non-exposed cohort

a) drawn from the same community as the exposed cohort *****

b) drawn from a different source

c) no description of the derivation of the non-exposed cohort

3. Ascertainment of exposure

a) secure record (e.g., surgical records) *****

b) structured interview *****

c) written self-report

d) no description

4. Demonstration that outcome of interest was not present at start of study

a) yes *****

b) no

**Comparability**

1. Comparability of cohorts on the basis of the design or analysis

a) study controls for age*

b) study controls for any additional factor (e.g., cognitive impairment, baseline comorbidities)*****

**Outcome**

1. Assessment of outcome

a) independent blind assessment*

b) record linkage*

c) self-report

d) no description

2. Was follow-up long enough for outcomes to occur

a) yes (follow-up ≥2 days)*

b) no

3. Adequacy of follow up of cohorts

a) complete follow up - all subjects accounted for*

b) subjects lost to follow up unlikely to introduce bias - small number lost - < 10% or description provided of those lost*

c) follow up rate <90% and no description of those lost

d) no statement

# eTable 5. Table of sensitivity analyses of prognostic factors associated with postoperative delirium among older adults undergoing elective surgery

| **Prognostic Factor** | **Number of Studies** | **Odds Ratio**  **(95% CI)** | **Heterogeneity (I^2^)** |
| --- | --- | --- | --- |
| History of Delirium | 0 | -- | -- |
| Frailty | 1 | -- | -- |
| Cognitive Impairment | 10 | 2.90 (1.59-5.26) | 78.87 |
| Renal Insufficiency | 2 | 2.04 (0.44-9.57) | 87.31 |
| Psychotropic Medication Use | 1 | 7.58 (1.53-37.46) | -- |
| Psychiatric History | 1 | 2.67 (1.22-5.83) | -- |
| Older Age | 9 | 2.05 (1.29-3.26) | 96.08 |
| ADL Impairment | 2 | 2.07 (1.38-3.10) | 0 |
| IADL Impairment | 0 | -- | -- |
| Cerebrovascular Disease | 2 | 2.43 (1.11-5.35) | 0 |
| Smoking Status | 1 | 10.5 (2.8-40.2) | -- |
| ASA Status | 2 | 3.42 (1.17-9.98) | 0 |
| Low Education | 0 | -- | -- |
| Diabetes Mellitus | 0 | -- | -- |
| Neurological Disease | 1 | 1.47 (1.01-2.13) | -- |
| Charlson Comorbidity Index | 0 | -- | -- |
| Caregiver Support | 0 | -- | -- |
| Male Sex | 2 | 1.15 (0.23-5.74) | 86.62 |
| Alcohol Consumption | 1 | 5.76 (1.52-21.84) | -- |
| Dyslipidemia | 0 | -- | -- |
| Hypertension | 2 | 3.13 (1.39-7.03) | 6.1 |
| Coronary Artery Disease | 0 | -- | -- |
| Myocardial Infarction | 0 | -- | -- |
| Obstructive Lung Disease | 0 | -- | -- |
| Hypoalbuminemia | 0 | -- | -- |
| Depression Scale Score | 0 | -- | -- |
| Greater Number of Drugs | 0 | -- | -- |
| Heart Failure | 0 | -- | -- |
| Body Mass Index (BMI) | 0 | -- | -- |
| General Anesthesia | 1 | 1.11 (0.26-4.71) | -- |
| Prehospitalization | 0 | -- | -- |
| **Abbreviations:** ADL – Activities of daily living; ASA – American Society of Anesthesiologists;  CI – Confidence interval; IADL – Instrumental activities of daily living | | | |

# eMethods 1. Data imputation methods

Where necessary, mean values and standard deviations were imputed from the median values and interquartile ranges by assuming the median value to be the same as the mean value of the study population and by dividing the interquartile range by 1.35 to approximate the standard deviation, assuming a normal distribution over the study population.^44^ These data imputation methods were applied to the following prognostic factors: age^23^, Charlson comorbidity index^3,42^, and body mass index (BMI)^37,42^.

If only 95% confidence intervals were available to represent the variance of a mean value, the standard deviation was estimated as per the following equation:^44^

*Standard deviation = √number of patients in the group x (upper bound of the confidence interval – lower bound of the confidence interval)/(appropriate value from t-distribution x 2)*

In the case of one study where only the standard error of the mean (SEM) was available, the standard deviation was estimated as per the following equation:^40,44^

*Standard deviation = standard error of the mean x √number of patients in the group*

# eMethods 2. Excluded study - explanation and data imputation methods trialed with examples

Tai S, Xu L, Zhang L, Fan S, and Liang C. Preoperative risk factors of postoperative delirium after transurethral prostatectomy for benign prostatic hyperplasia. Int J Clin Exp Med 2015;8(3):4569-4574.

We attempted to contact the corresponding author from this paper on two occasions without receiving any response (August 29, 2016, and September 8, 2016).


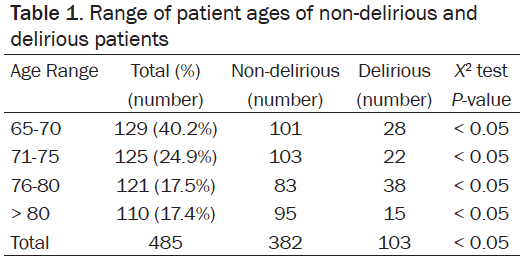


This is Table 1 from Tai et al., 2015. Despite having 17.4% of the total patients >80 years of age, the mean (standard deviation) is reported as 71.25+/-2.35.

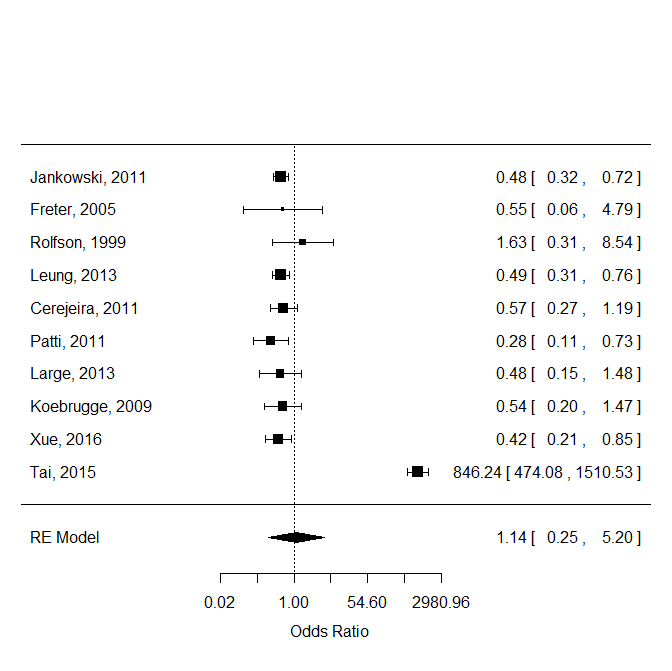


**Figure 1.** Forest plot of the individual effect measures and the summary effect measure for the odds of postoperative delirium in a patient with better baseline functional impairment in activities of daily living (I^2^=97.72%).


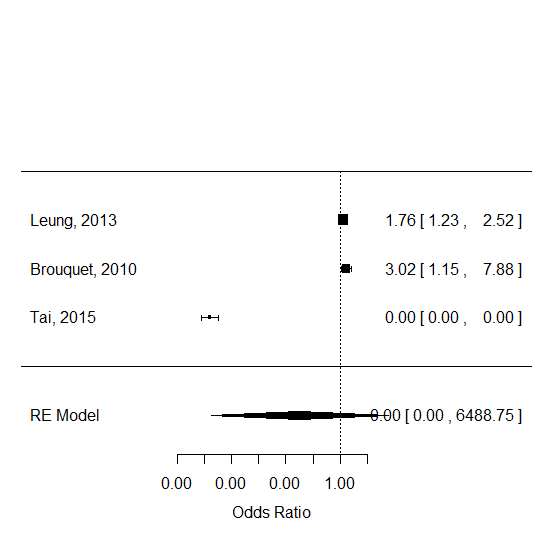


**Figure 2.** Forest plot of the individual effect measures and the summary effect measure for the odds of postoperative delirium in a patient with worse baseline functional impairment in instrumental activities of daily living (I^2^=99.90%).

It would not be correct to report mean +/- standard deviations when you have used a Mann-Whitney U test in which you are assuming the populations to be non-normally distributed.

Assuming the reported variances in Table 4 are in fact standard deviations, the mean difference between the two groups would be 5.52 (95% CI 5.42-5.62, p<0.0001), which is much more highly significant than the reported p-value.

Next, assuming the mean values for the delirious and non-delirious groups as well as the p-value for the Mann-Whitney U test reported are correct, we then tried to estimate a value for the standard deviation of each population using the method suggested in the Cochrane Handbook, Section 7.7.3.3.^44^ To use this method, we had to assume that the p-value from the Mann-Whitney U test was similar to that of a t-test. Using this method, the estimated standard deviation would be 21.3, which is impossible because the Geriatric Depression Scale is a Likert scale with values from 0-15.

Alternatively, if we assumed the variance measure reported was in fact a standard error as opposed to a standard deviation, the estimated standard deviation would then be 2.94 for the group of patients with delirium and 15.05 for the group of patients without delirium. Again, this value for the group of patients without delirium is impossible given the nature of the Geriatric Depression Scale.

Neither in the text, nor in the table do the authors state what scales were used to measure the ADLs and IADLs; therefore, it was not possible to estimate a variance based on the variance of similarly reported outcomes in other papers.

For these aforementioned reasons, it was decided to exclude the paper by Tai et al., 2015, from the meta-analyses in our manuscript.

# eFigure 1. Forest plots of study-level and overall effect estimates for prognostic factors associated with postoperative delirium among older adults undergoing elective surgery


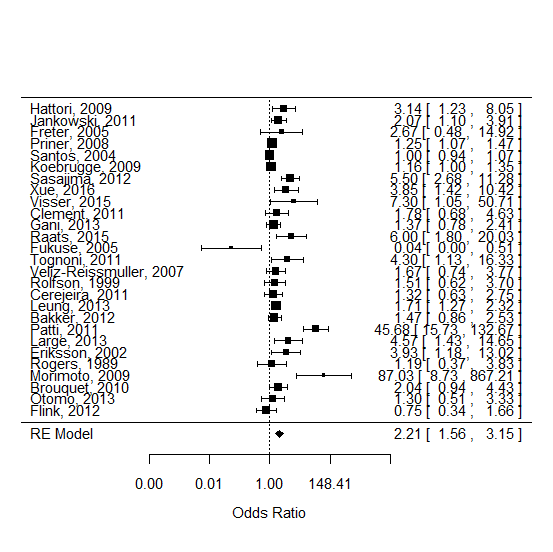


**Figure 1a.** Forest plot of the study-level effect measures and the summary effect measure for the odds of postoperative delirium in an older patient (I^2^=94.82%).


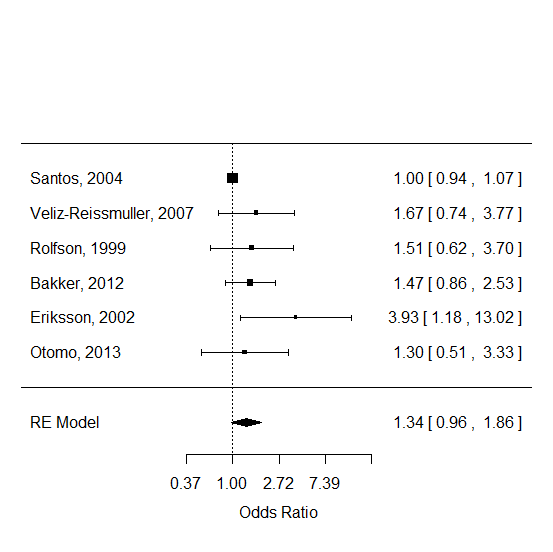


**Figure 1b.** Forest plot of the study-level effect measures and the summary effect measure for the odds of postoperative delirium in an older patient undergoing cardiac surgery (I^2^=44.14%).


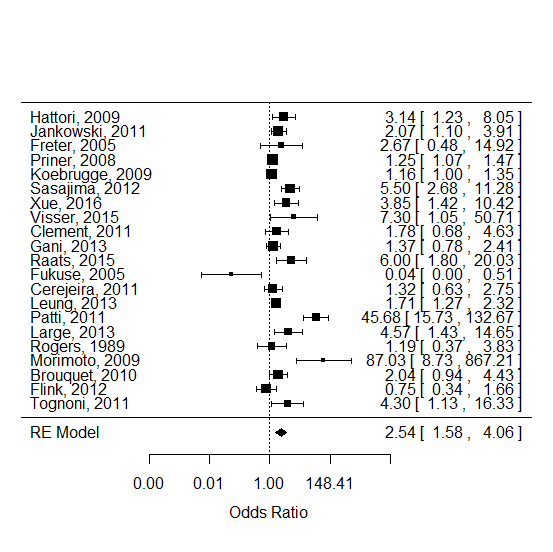


**Figure 1c.** Forest plot of the study-level effect measures and the summary effect measure for the odds of postoperative delirium in an older patient undergoing non-cardiac surgery (I^2^=93.59%).


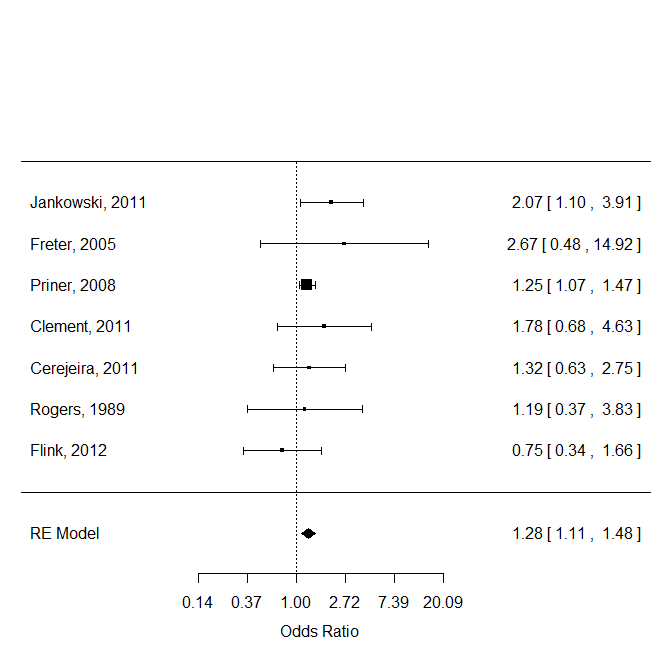


**Figure 1d.** Forest plot of the study-level effect measures and the summary effect measure for the odds of postoperative delirium in an older patient undergoing orthopedic surgery (I^2^=0%).


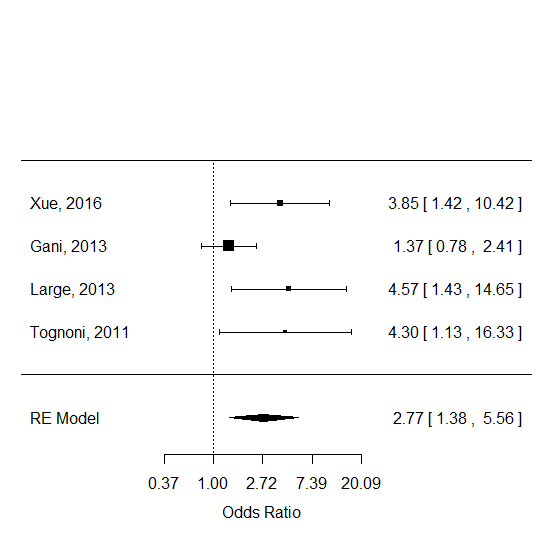


**Figure 1e.** Forest plot of the study-level effect measures and the summary effect measure for the odds of postoperative delirium in an older patient undergoing urological surgery (I^2^=51.92%).


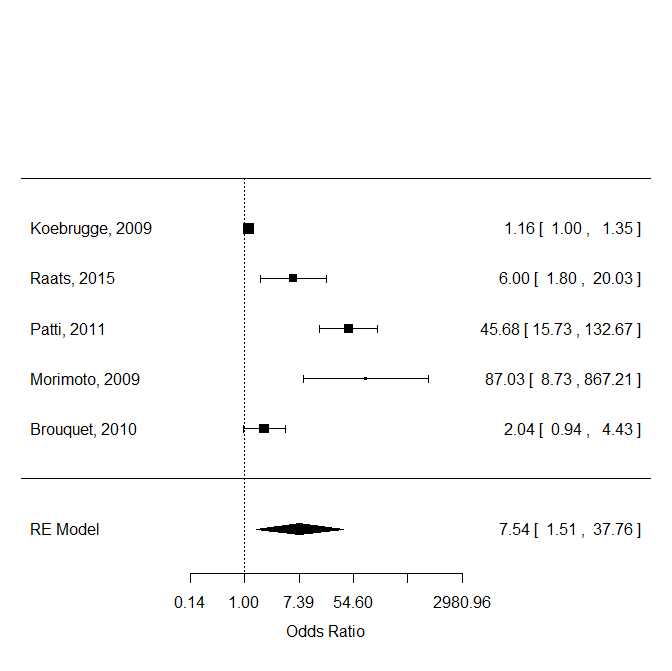


**Figure 1f.** Forest plot of the study-level effect measures and the summary effect measure for the odds of postoperative delirium in an older patient undergoing abdominal surgery (I^2^=94.93%).


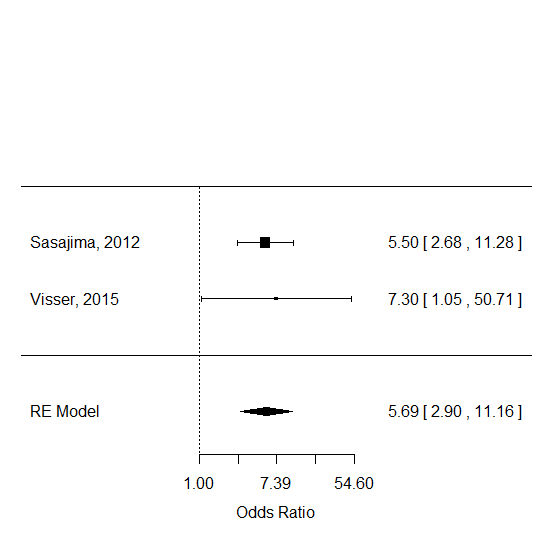


**Figure 1g.** Forest plot of the study-level effect measures and the summary effect measure for the odds of postoperative delirium in an older patient undergoing vascular surgery (I^2^=0%).


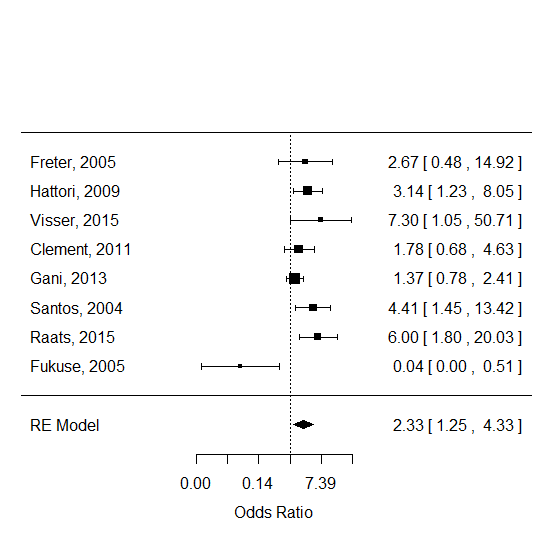


**Figure 1h**. Forest plot of the study-level effect measures and the summary effect measure for the odds of postoperative delirium in a patient aged 80 years of age or older (I^2^=56.09%).


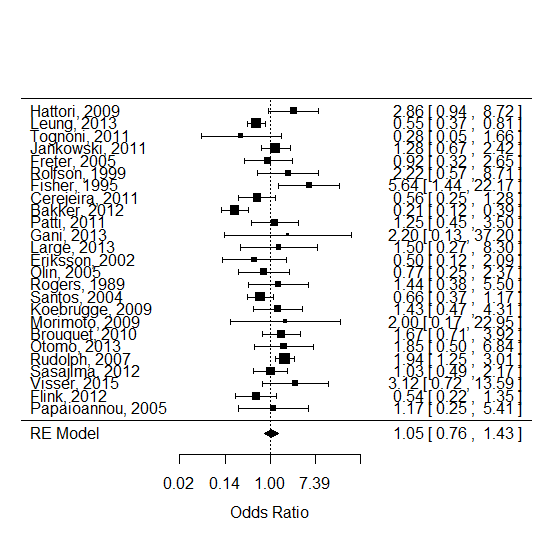


**Figure 2.** Forest plot of the study-level effect measures and the summary effect measure for the odds of postoperative delirium in a male patient compared to a female patient (I^2^=62.61%).


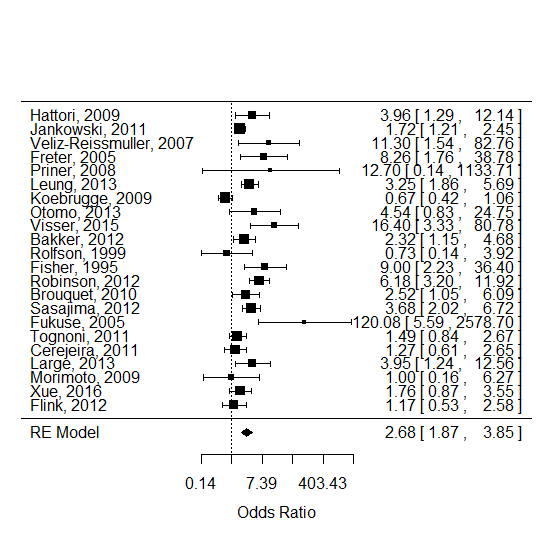


**Figure 3a.** Forest plot of the study-level effect measures and the summary effect measure for the odds of postoperative delirium in a patient with cognitive impairment (I^2^=72.86%).


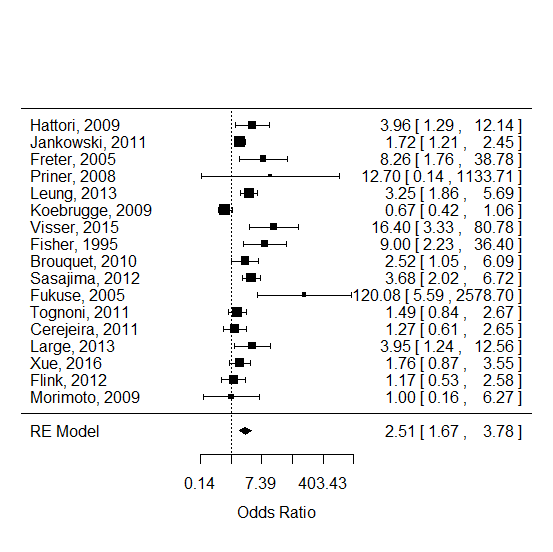


**Figure 3b.** Forest plot of the study-level effect measures and the summary effect measure for the odds of postoperative delirium in a patient with cognitive impairment undergoing non-cardiac surgery (I^2^=74.96%).


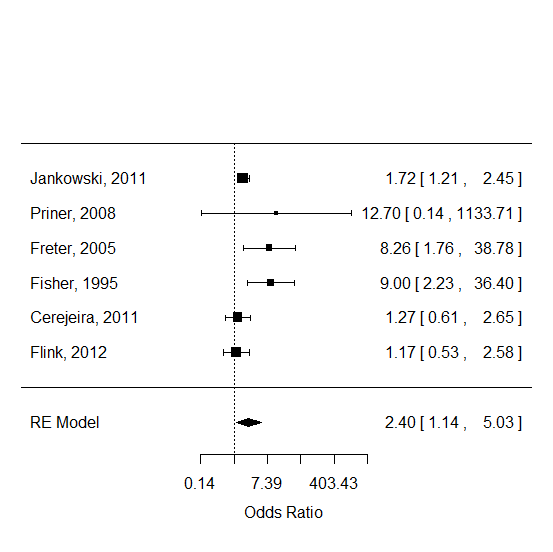


**Figure 3c.** Forest plot of the study-level effect measures and the summary effect measure for the odds of postoperative delirium in a patient with cognitive impairment undergoing orthopedic surgery (I^2^=72.20%).


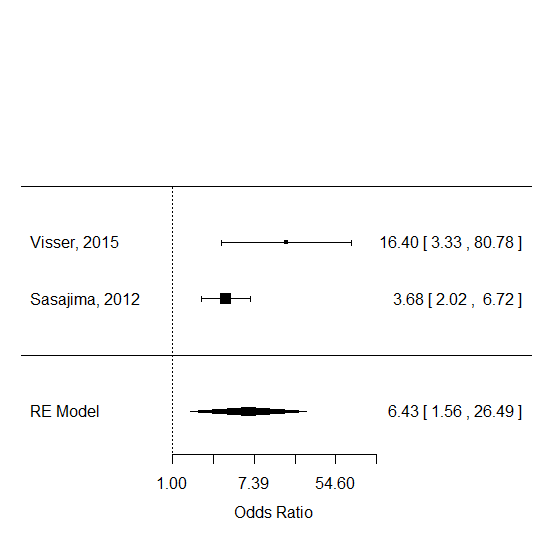


**Figure 3d**. Forest plot of the study-level effect measures and the summary effect measure for the odds of postoperative delirium in a patient with cognitive impairment undergoing vascular surgery (I^2^=66.13%).


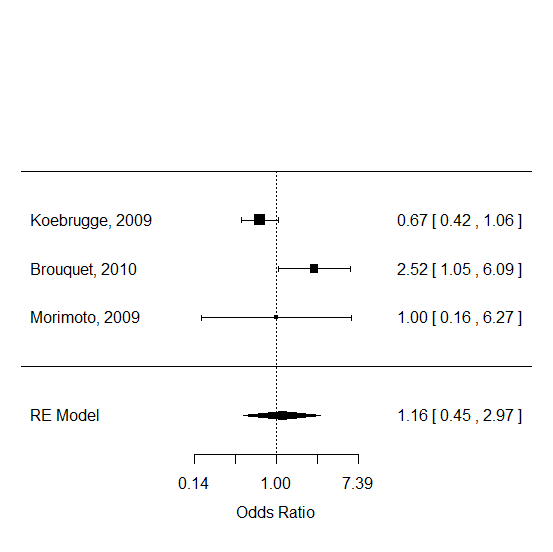


**Figure 3e.** Forest plot of the study-level effect measures and the summary effect measure for the odds of postoperative delirium in a patient with cognitive impairment undergoing abdominal surgery (I^2^=67.99%).


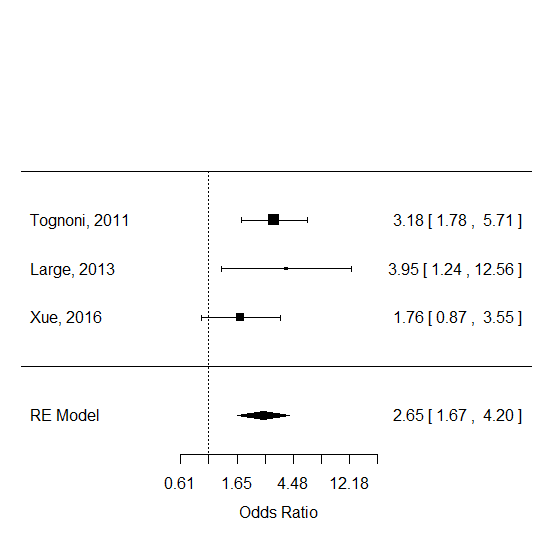


**Figure 3f.** Forest plot of the study-level effect measures and the summary effect measure for the odds of postoperative delirium in a patient with cognitive impairment undergoing urological surgery (I^2^=13.65%).


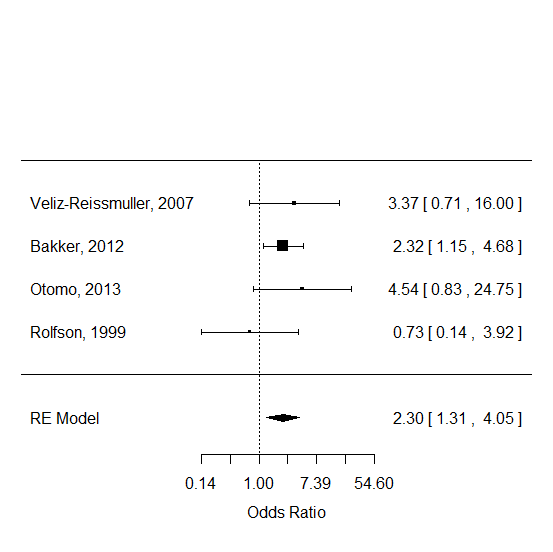


**Figure 3g.** Forest plot of the study-level effect measures and the summary effect measure for the odds of postoperative delirium in a patient with cognitive impairment undergoing cardiac surgery (I^2^=0%).


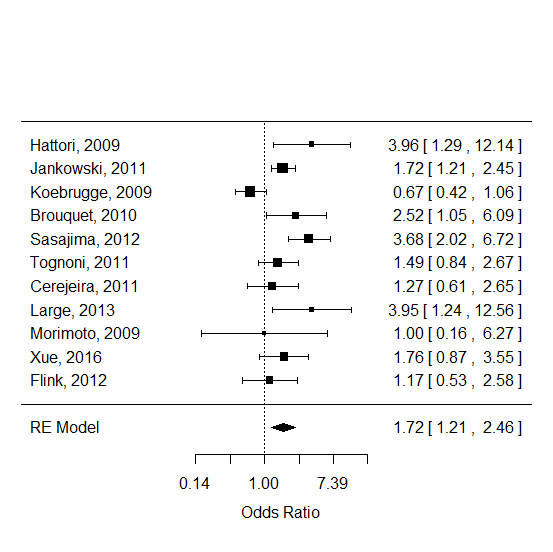


**Figure 3h.** Forest plot of the study-level effect measures and the summary effect measure for the odds of postoperative delirium in a patient with cognitive impairment (studies where cognitive impairment was an exclusion criteria are excluded from analysis; I^2^=64.61%).


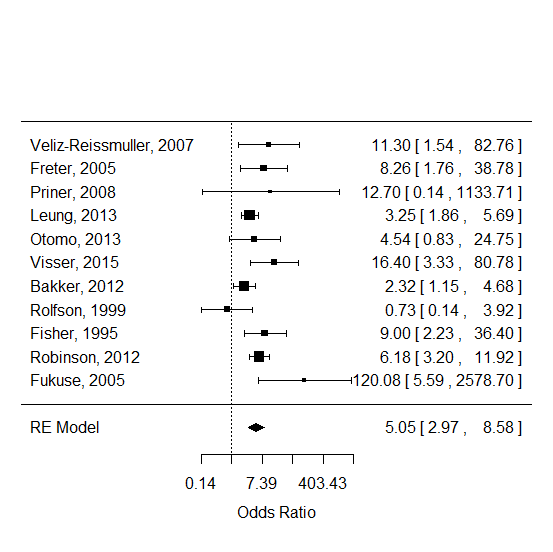


**Figure 3i.** Forest plot of the study-level effect measures and the summary effect measure for the odds of postoperative delirium in a patient with cognitive impairment (studies where cognitive impairment was not an exclusion criteria; I^2^=48.59%).


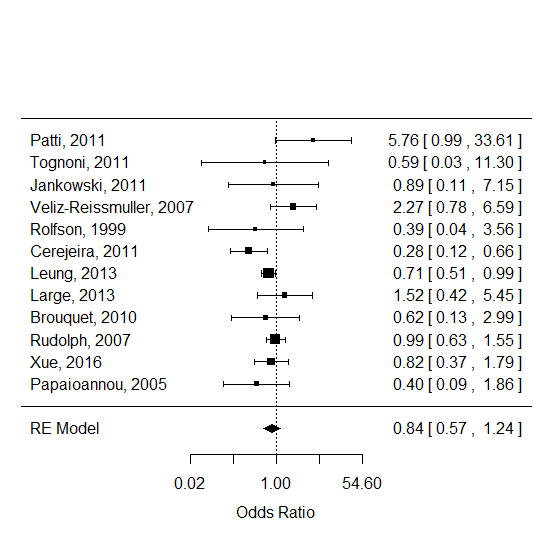


**Figure 4a.** Forest plot of the study-level effect measures and the summary effect measure for the odds of postoperative delirium in a patient with alcohol consumption (I^2^=45.13%).


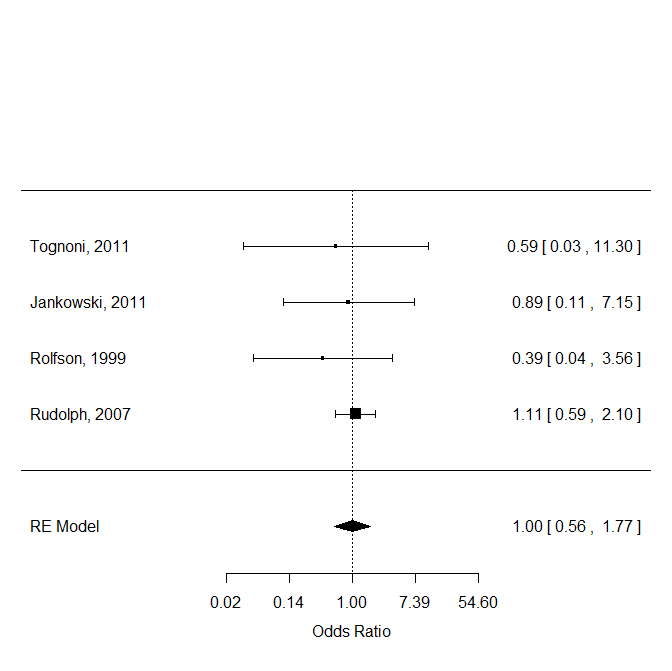


**Figure 4b.** Forest plot of the study-level effect measures and the summary effect measure for the odds of postoperative delirium in a patient with alcohol abuse (I^2^=0%).


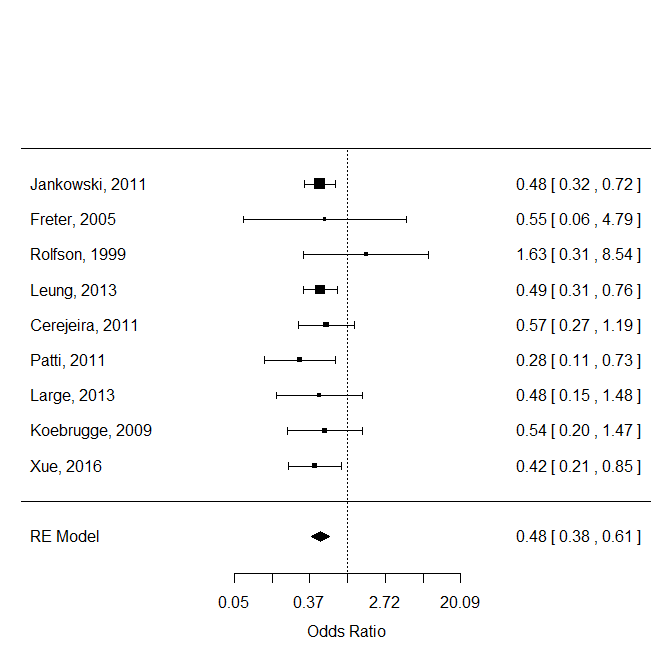


**Figure 5.** Forest plot of the study-level effect measures and the summary effect measure for the odds of postoperative delirium in a patient without baseline functional impairment in activities of daily living (I^2^=0%).


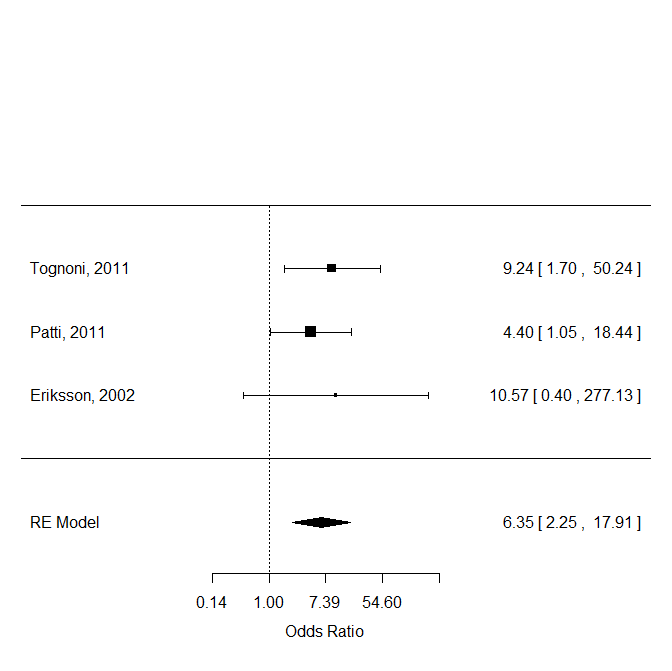


**Figure 6.** Forest plot of the study-level effect measures and the summary effect measure for the odds of postoperative delirium in a patient with a history of delirium (I^2^=0%).


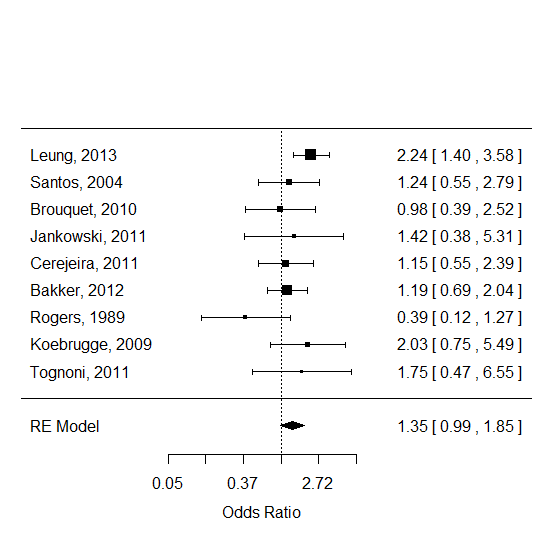


**Figure 7.** Forest plot of the study-level effect measures and the summary effect measure for the odds of postoperative delirium in patients with a greater depression screen score (I^2^=25.55%).


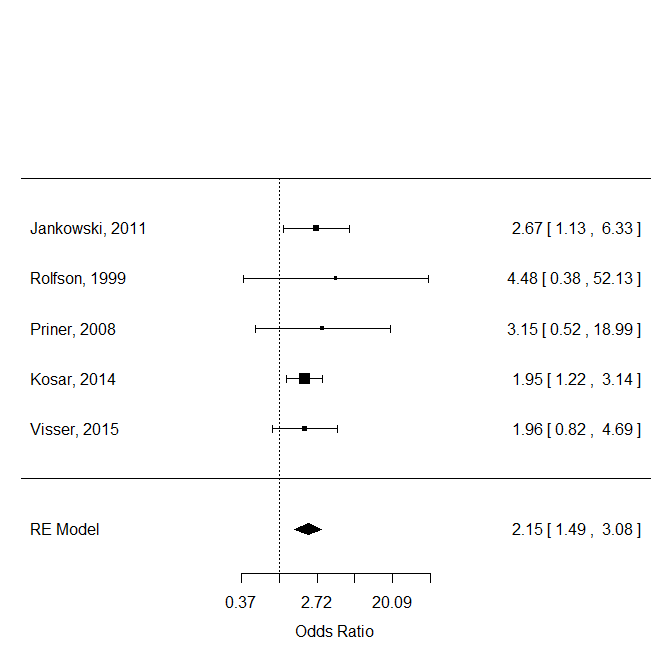


**Figure 8.** Forest plot of the study-level effect measures and the summary effect measure for the odds of postoperative delirium in a patient with a psychiatric history (I^2^=0%).


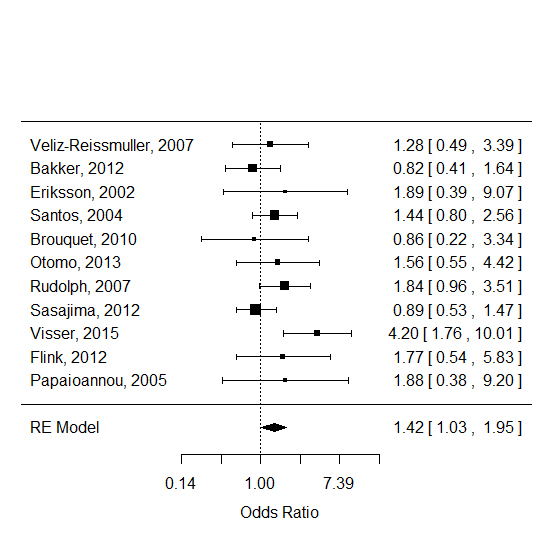


**Figure 9.** Forest plot of the study-level effect measures and the summary effect measure for the odds of postoperative delirium in a patient with a history of diabetes mellitus (I^2^=36.64%).


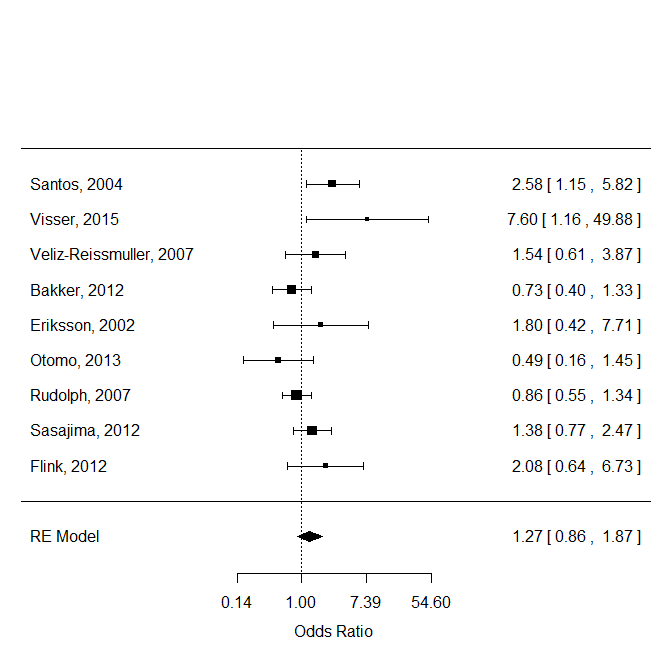


**Figure 10.** Forest plot of the study-level effect measures and the summary effect measure for the odds of postoperative delirium in a patient with a history of hypertension (I^2^=48.89%).


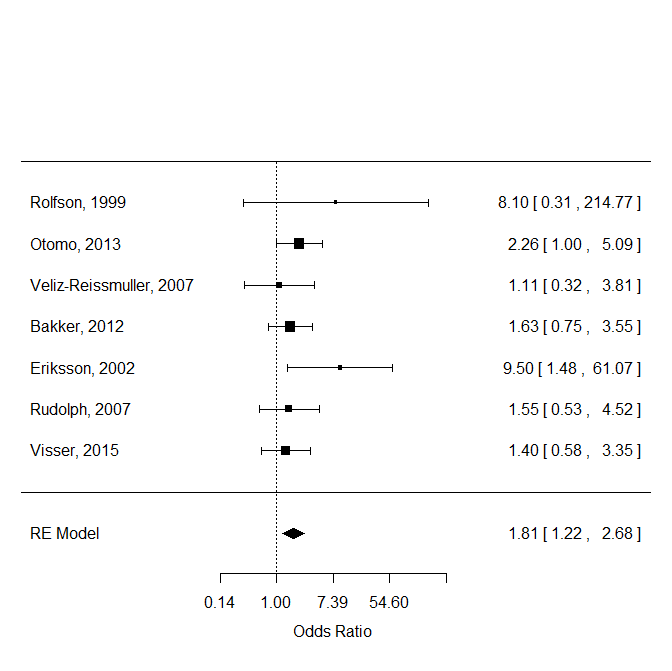


**Figure 11.** Forest plot of the study-level effect measures and the summary effect measure for the odds of postoperative delirium in a patient with a history of cerebrovascular disease (I^2^=0%).


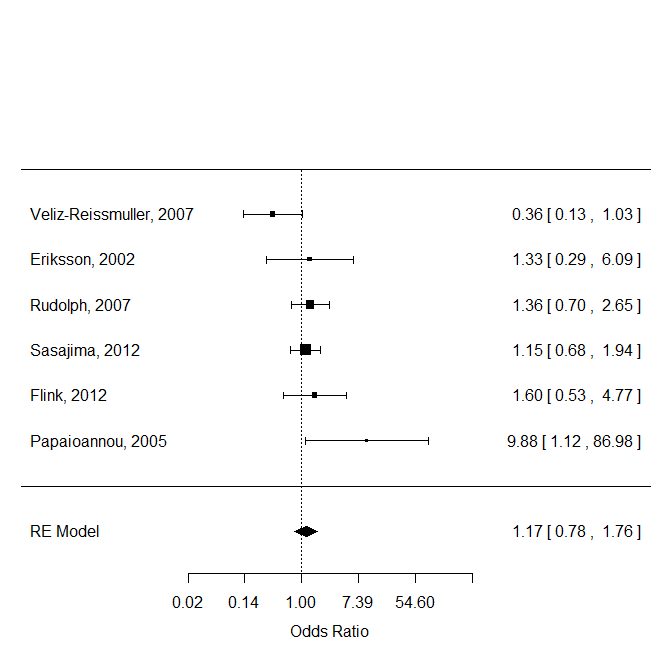


**Figure 12.** Forest plot of the study-level effect measures and the summary effect measure for the odds of postoperative delirium in a patient with a history of coronary artery disease (I^2^=17.00%).


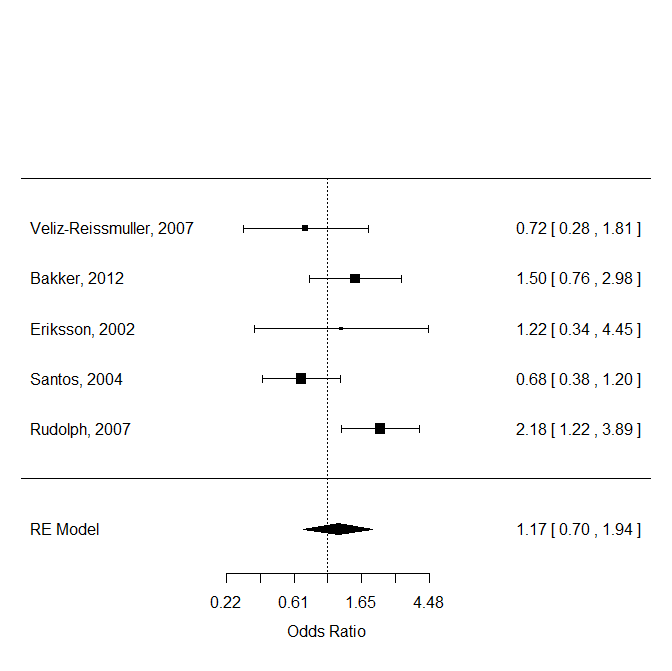


**Figure 13.** Forest plot of the study-level effect measures and the summary effect measure for the odds of postoperative delirium in a patient with a history of myocardial infarction (I^2^=56.70%).


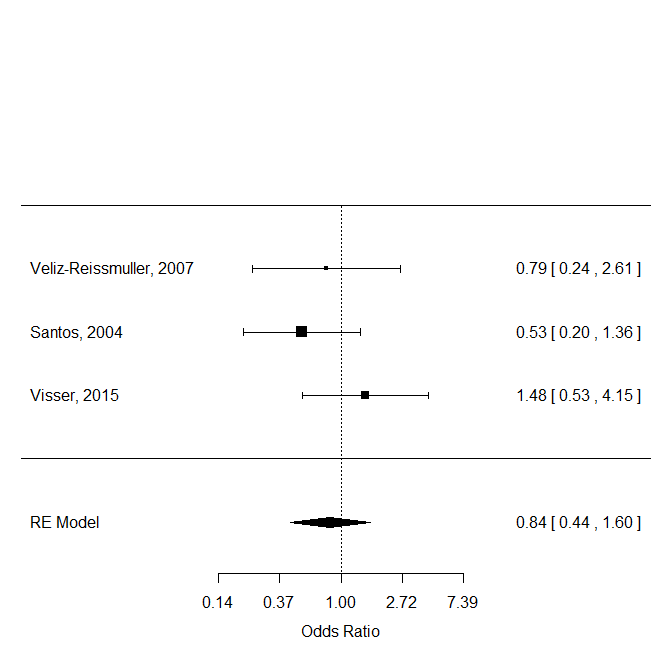


**Figure 14.** Forest plot of the study-level effect measures and the summary effect measure for the odds of postoperative delirium in a patient with a history of obstructive lung disease (I^2^=13.24%).


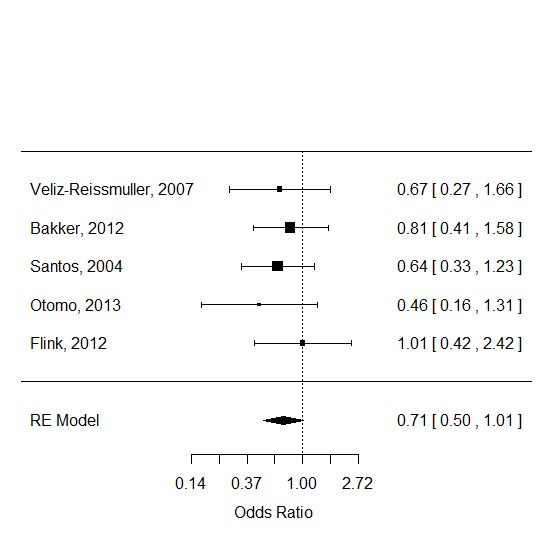


**Figure 15.** Forest plot of the study-level effect measures and the summary effect measure for the odds of postoperative delirium in a patient with a history of dyslipidemia (I^2^=0%).


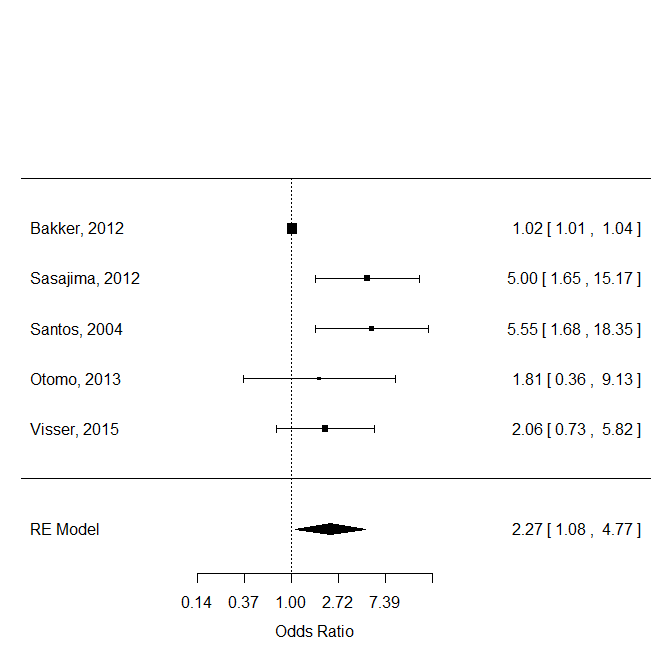


**Figure 16a.** Forest plot of the study-level effect measures and the summary effect measure for the odds of postoperative delirium in a patient with a history of renal insufficiency (I^2^=70.91%).


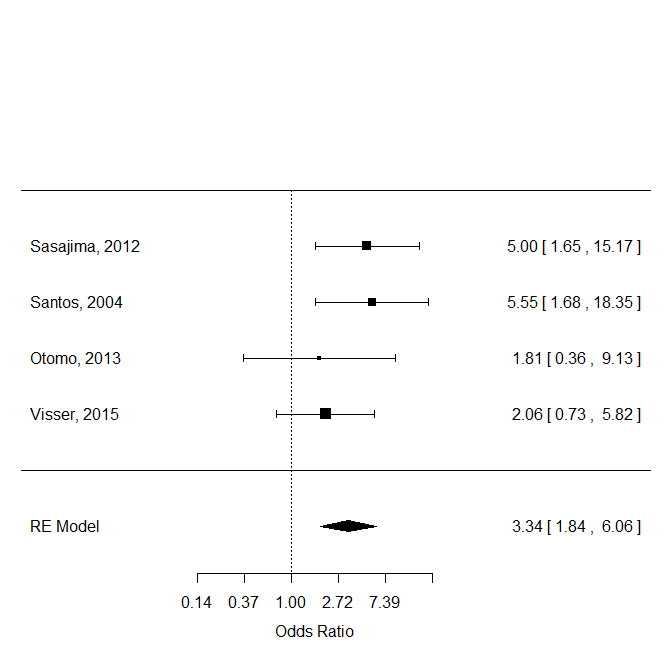


**Figure 16b.** Forest plot of the study-level effect measures and the summary effect measure for the odds of postoperative delirium in a patient with a history of renal insufficiency (Bakker 2012 removed - creatinine level as opposed to history of kidney disease, I^2^=0%).


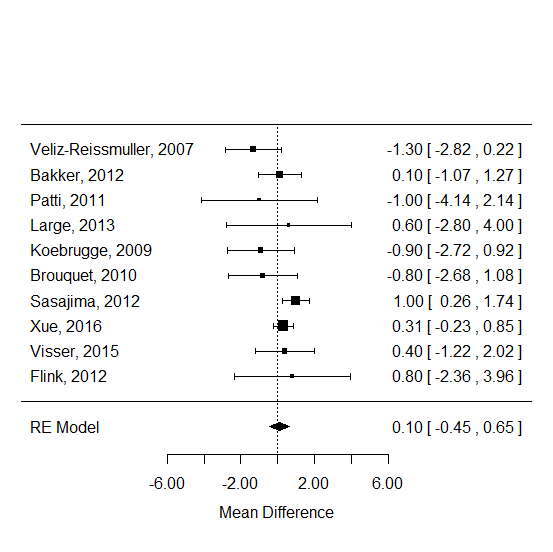


**Figure 17.** Forest plot of the study-level effect measures and the summary effect measure for the mean difference in BMI between patients with and without postoperative delirium (I^2^=36.12%).


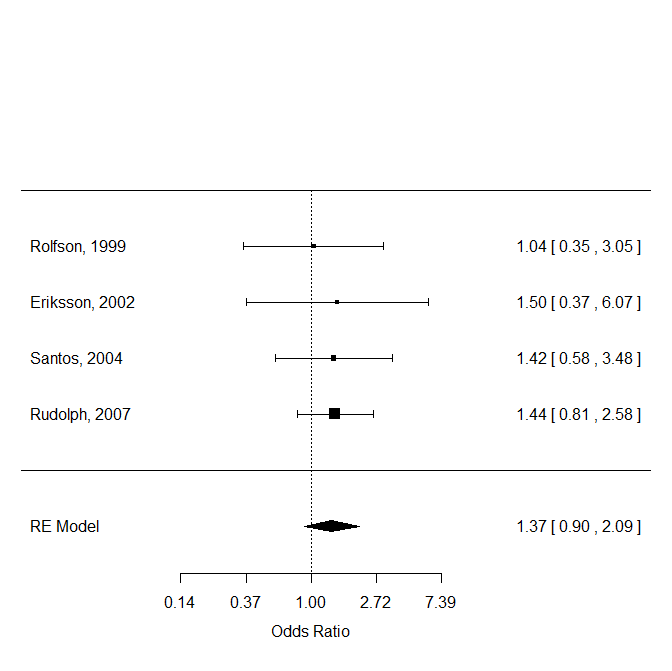


**Figure 18.** Forest plot of the study-level effect measures and the summary effect measure for the odds of postoperative delirium in a patient with a history of heart failure (I^2^=0%).


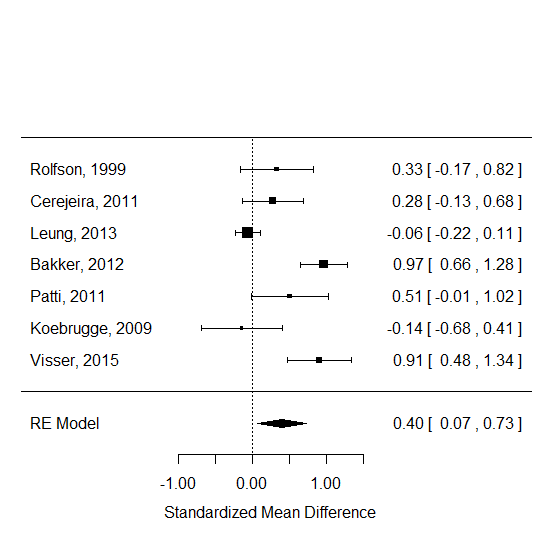


**Figure 19a.** Forest plot of the study-level effect measures and the summary effect measure for the mean difference in Charlson Comorbidity Index between patients with and without postoperative delirium (I^2^=82.52%).


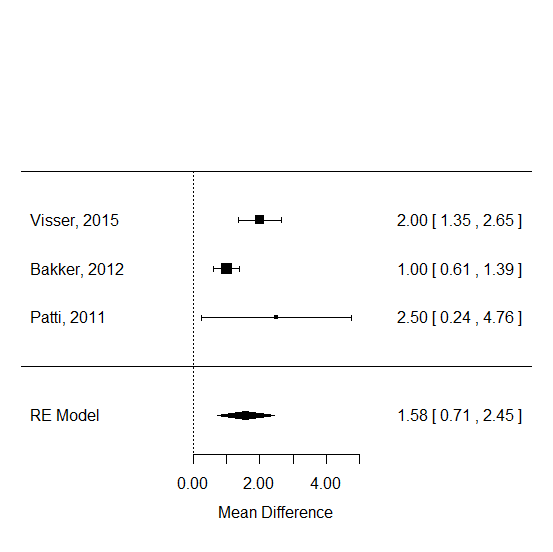


**Figure 19b.** Forest plot of the study-level effect measures and the summary effect measure for the mean difference in Charlson Comorbidity Index (age included in score) between patients with and without postoperative delirium (I^2^=73.22%).


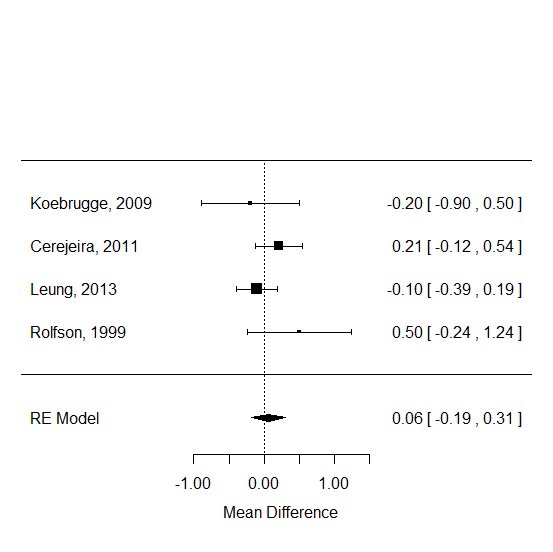


**Figure 19c.** Forest plot of the study-level effect measures and the summary effect measure for the mean difference in Charlson Comorbidity Index (without age included in score) between patients with and without postoperative delirium (I^2^=22.68%).


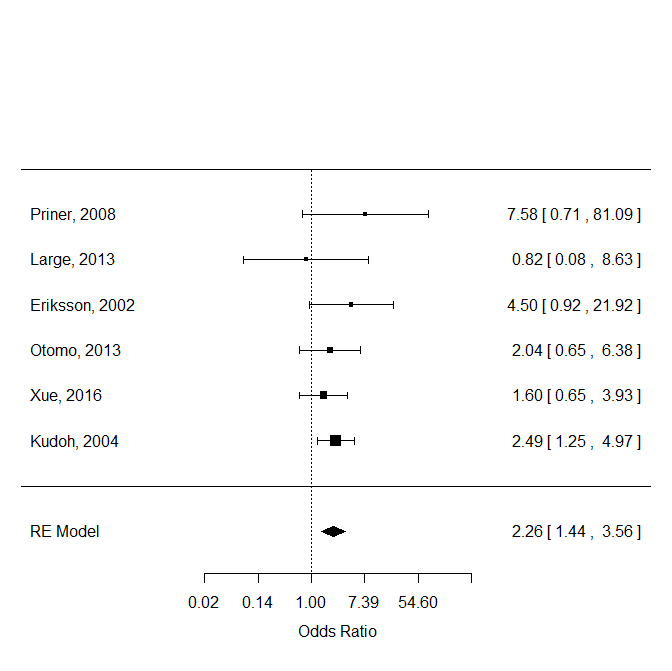


**Figure 20.** Forest plot of the study-level effect measures and the summary effect measure for the odds of postoperative delirium in a patient with a history of psychotropic medication use (I^2^=0%).


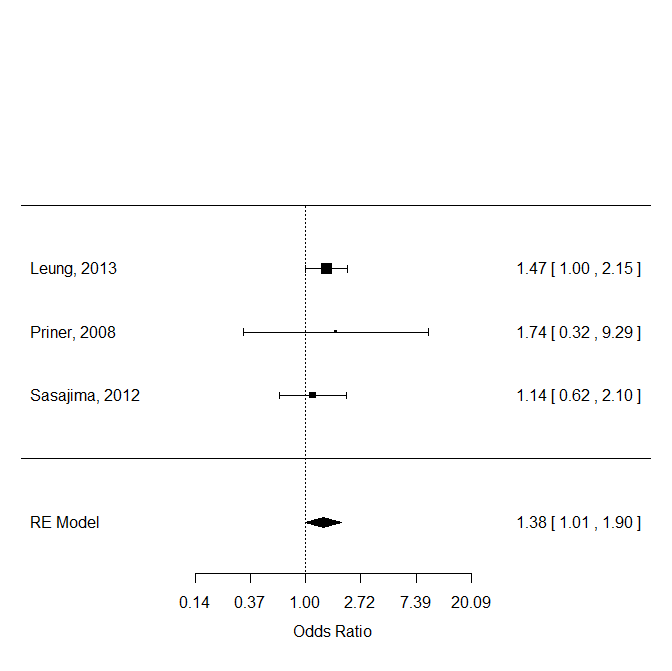


**Figure 21.** Forest plot of the study-level effect measures and the summary effect measure for the odds of postoperative delirium in a patient with a history of neurological disease (I^2^=0%).


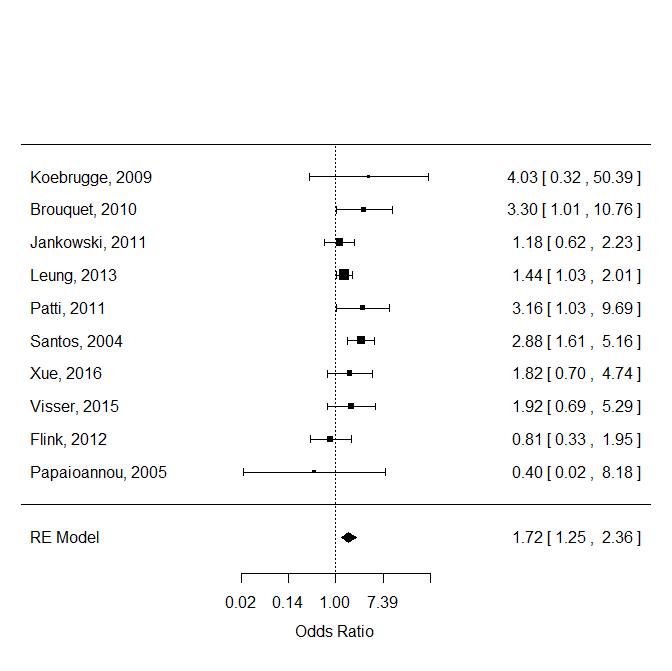


**Figure 22**. Forest plot of the study-level effect measures and the summary effect measure for the odds of postoperative delirium in a patient with an elevated American Society of Anesthesiologists (ASA) score (I^2^=29.33%).


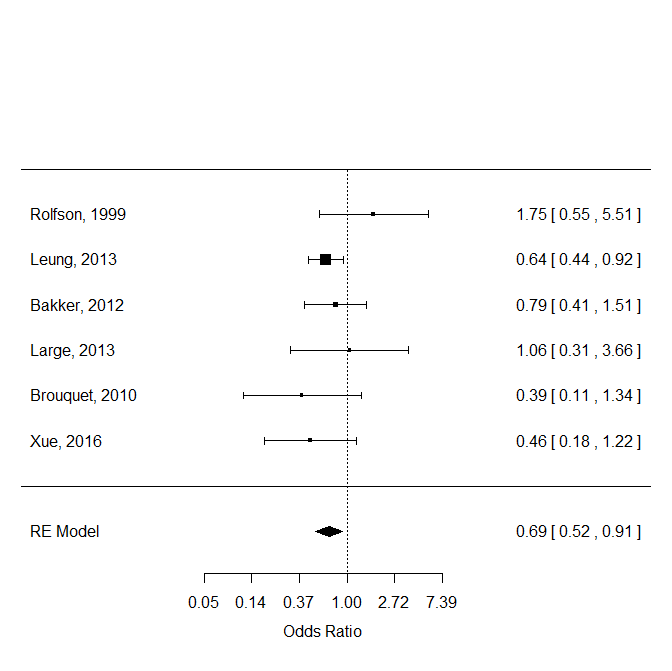


**Figure 23.** Forest plot of the study-level effect measures and the summary effect measure for the odds of postoperative delirium in a patient with availability of caregiver support (I^2^=0%).


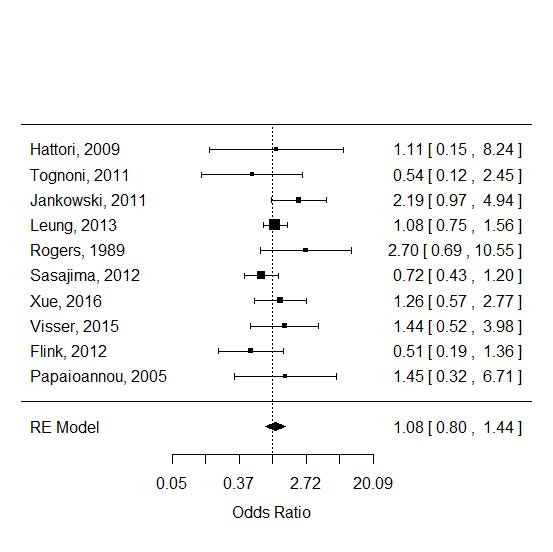


**Figure 24.** Forest plot of the study-level effect measures and the summary effect measure for the odds of postoperative delirium in a patient with general anesthesia during elective surgery (I^2^=19.62%).


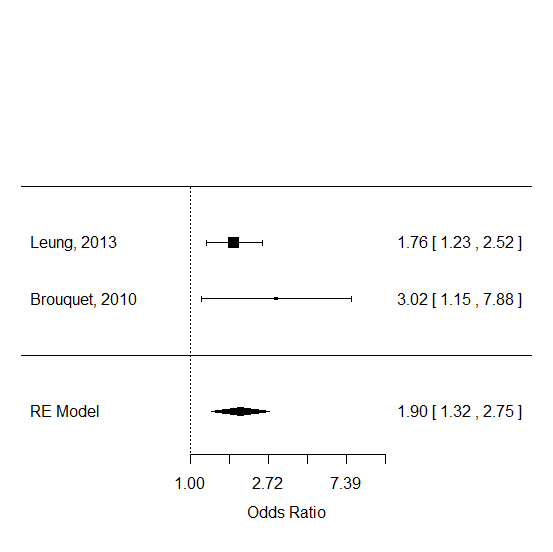


**Figure 25.** Forest plot of the study-level effect measures and the summary effect measure for the odds of postoperative delirium in a patient with baseline functional impairment in instrumental activities of daily living (I^2^=5.18%).


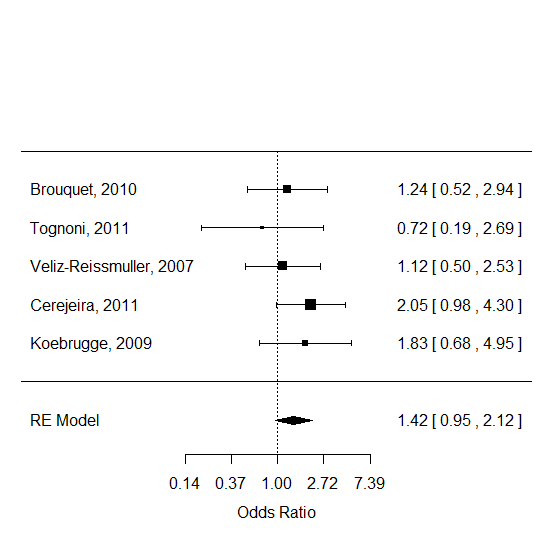


**Figure 26.** Forest plot of the study-level effect measures and the summary effect measure for the odds of postoperative delirium in a patient with greater numbers of medications prescribed preoperatively (I^2^=0%).


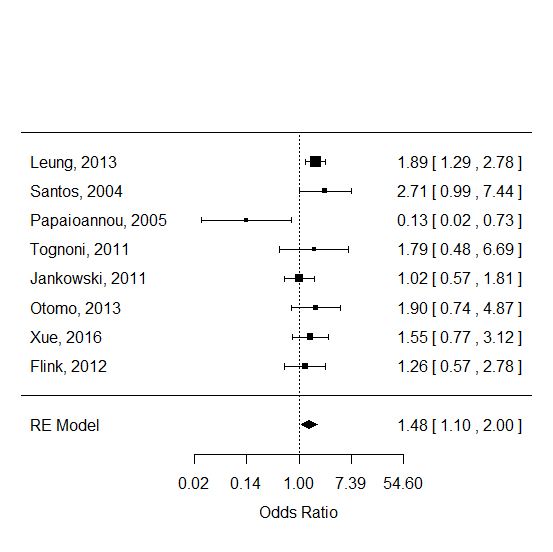


**Figure 27.** Forest plot of the study-level effect measures and the summary effect measure for the odds of postoperative delirium in a patient with lower education (I^2^=19.60%).


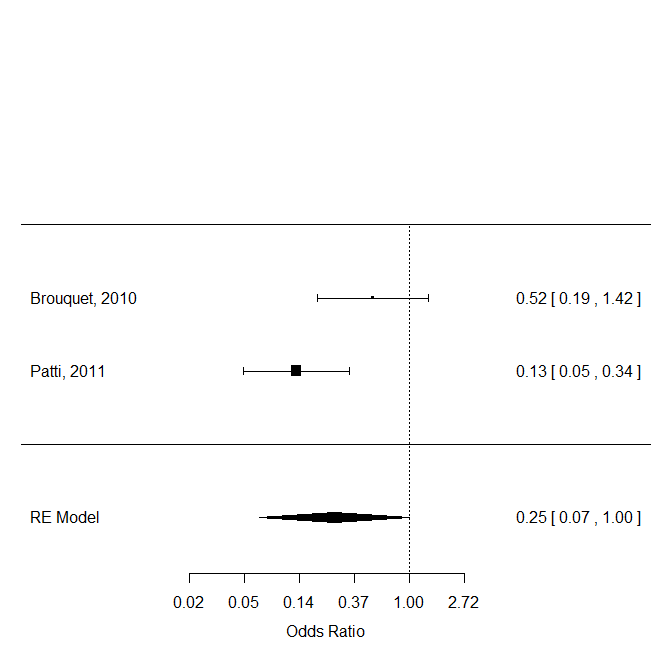


**Figure 28.** Forest plot of the study-level effect measures and the summary effect measure for the odds of postoperative delirium in an older with normal serum albumin (I^2^=73.64%).


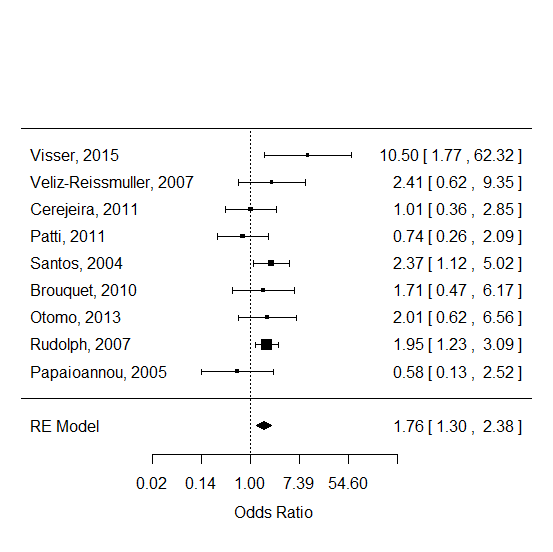


**Figure 29a.** Forest plot of the study-level effect measures and the summary effect measure for the odds of postoperative delirium in a smoker (I^2^=0%).


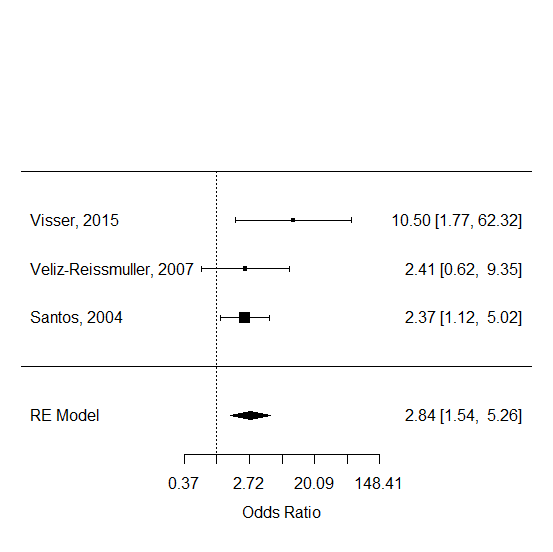


**Figure 29b.** Forest plot of the study-level effect measures and the summary effect measure for the odds of postoperative delirium in a current smoker (I^2^=0%).


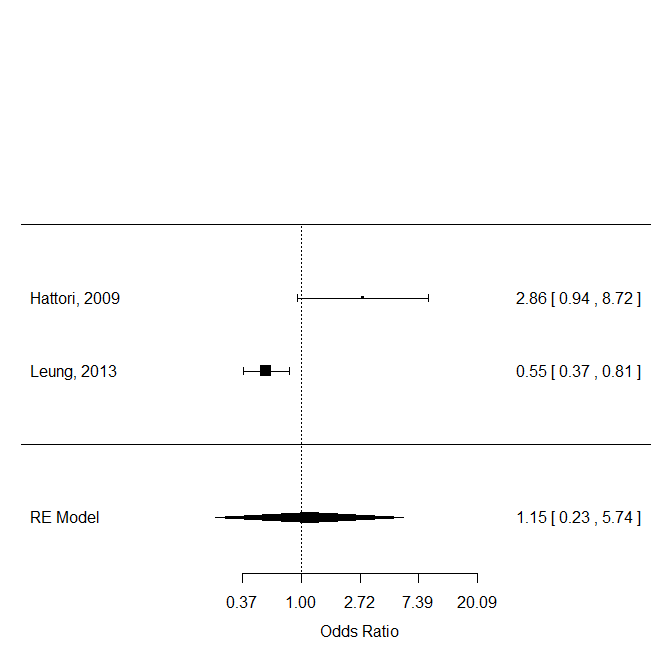


**Figure 30.** Forest plot of the study-level adjusted effect measures and the summary adjusted effect measure for the odds of postoperative delirium in a male patient (I^2^=86.62%).


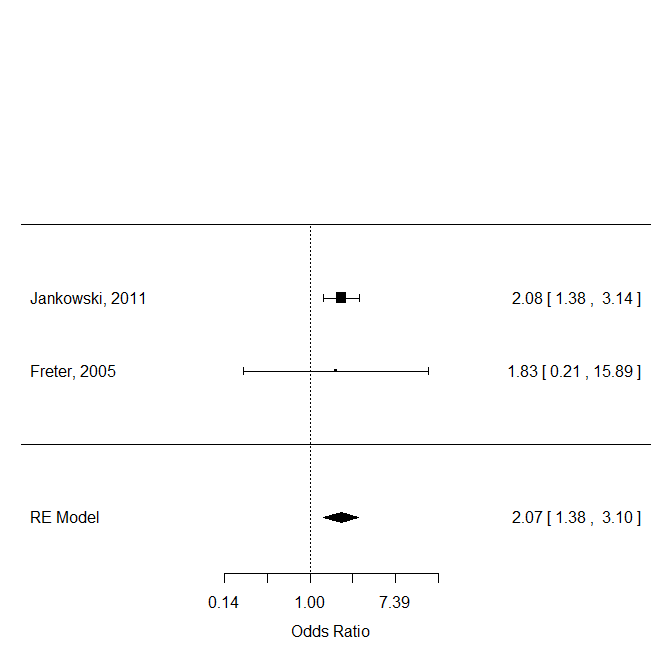


**Figure 31.** Forest plot of the study-level adjusted effect measures and the summary adjusted effect measure for the odds of postoperative delirium in a patient with greater impairment in activities of daily living (I^2^=0%).


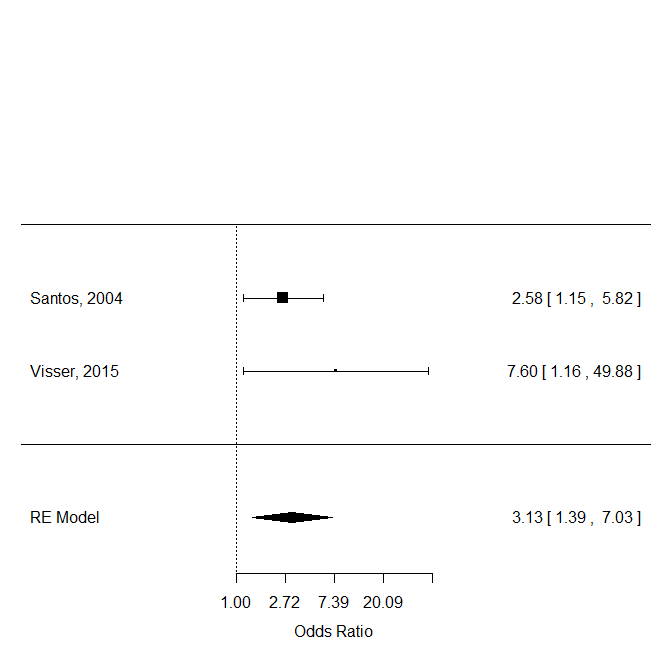


**Figure 32.** Forest plot of the study-level adjusted effect measures and the summary adjusted effect measure for the odds of postoperative delirium in a patient with hypertension (I^2^=6.10%).


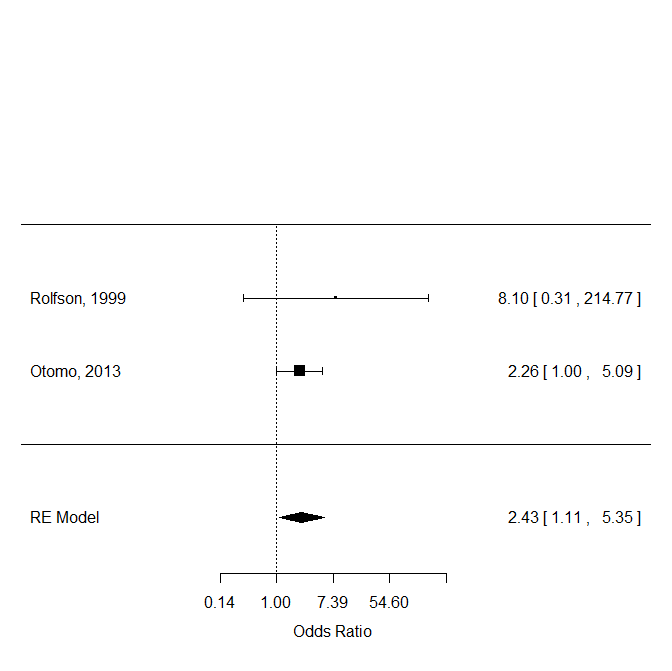


**Figure 33.** Forest plot of the study-level adjusted effect measures and the summary adjusted effect measure for the odds of postoperative delirium in a patient with cerebrovascular disease (I^2^=0%).


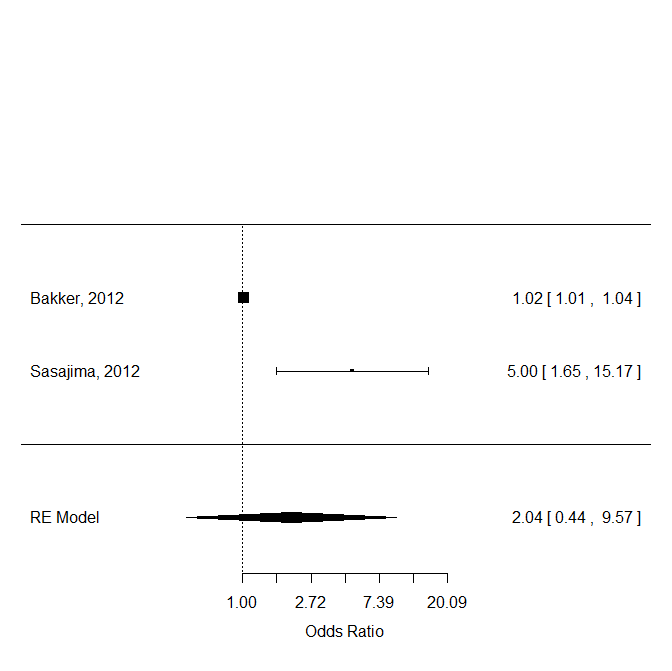


**Figure 34.** Forest plot of the study-level adjusted effect measures and the summary adjusted effect measure for the odds of postoperative delirium in a patient with renal insufficiency (I^2^=87.31%).


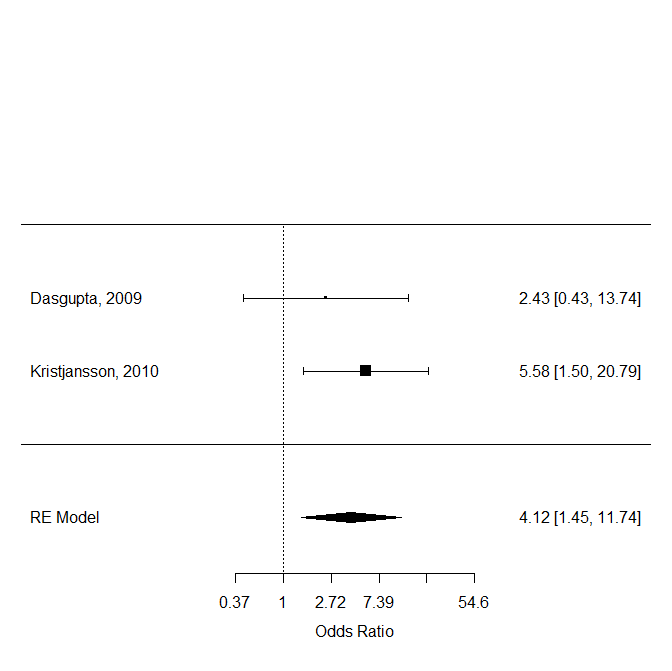


**Figure 35.** Forest plot of the study-level adjusted effect measures and the summary adjusted effect measure for the odds of postoperative delirium in a patient with frailty (I^2^=0%).


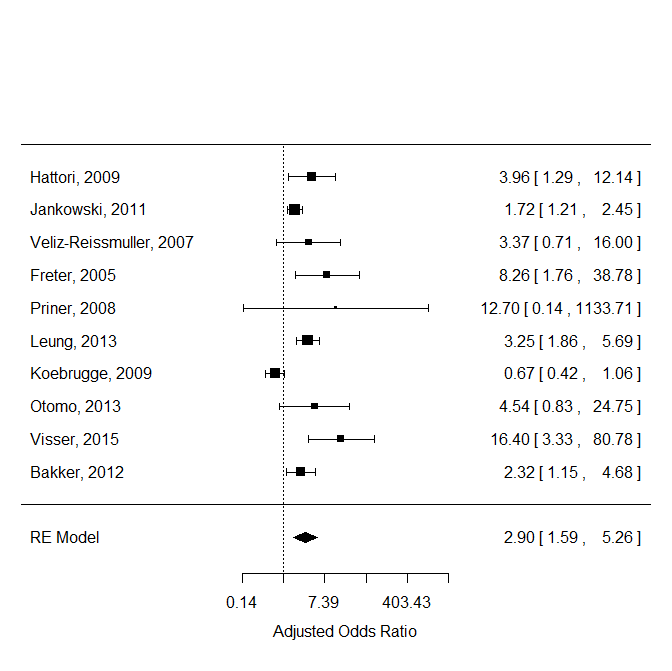


**Figure 36a.** Forest plot of the study-level adjusted effect measures and the summary adjusted effect measure for the odds of postoperative delirium in a patient with cognitive impairment (I^2^=78.87%).


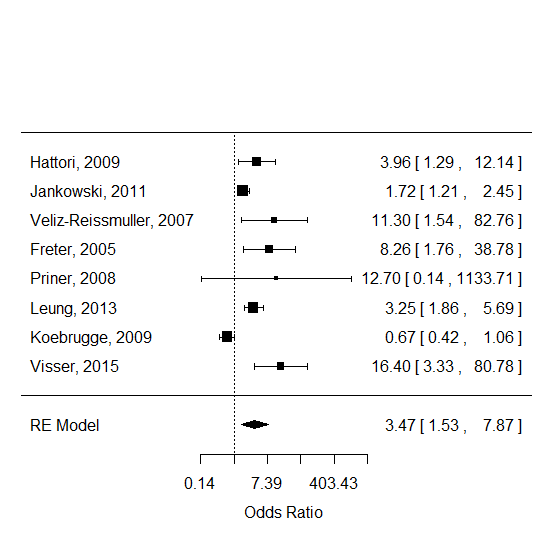


**Figure 36b.** Forest plot of the study-level effect measures adjusted for age and the summary adjusted effect measure for the odds of postoperative delirium in a patient with cognitive impairment (I^2^=86.53%).


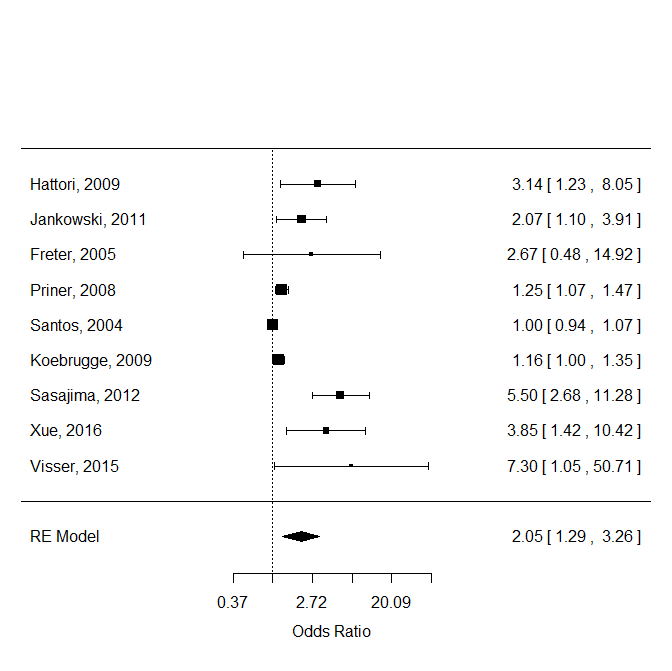


**Figure 37.** Forest plot of the study-level adjusted effect measures and the summary adjusted effect measure for the odds of postoperative delirium in an older patient (I^2^=96.08%).


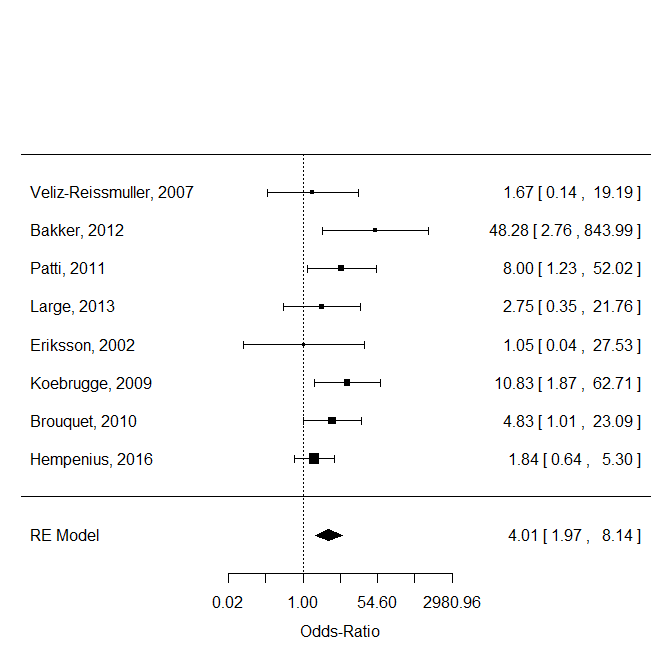


**Figure 38.** Forest plot of the study-level effect measures and the summary effect measure for the odds of postoperative mortality in patients with postoperative delirium (I^2^=14.7%).


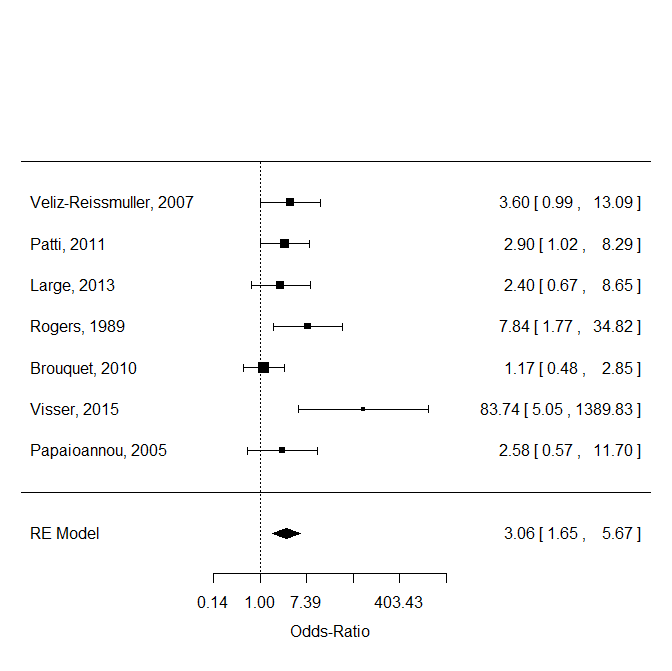


**Figure 39.** Forest plot of the study-level effect measures and the summary effect measure for the odds of postoperative complications in patients with postoperative delirium (I^2^=35.05%).


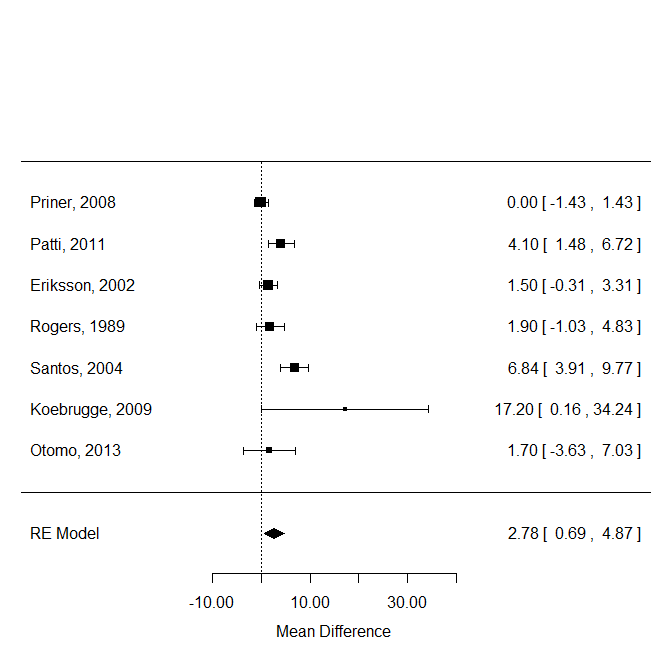


**Figure 40.** Forest plot of the study-level effect measures and the summary effect measure for the length of hospitalization in patients with postoperative delirium compared to those patients without postoperative delirium (I^2^=73.72%).


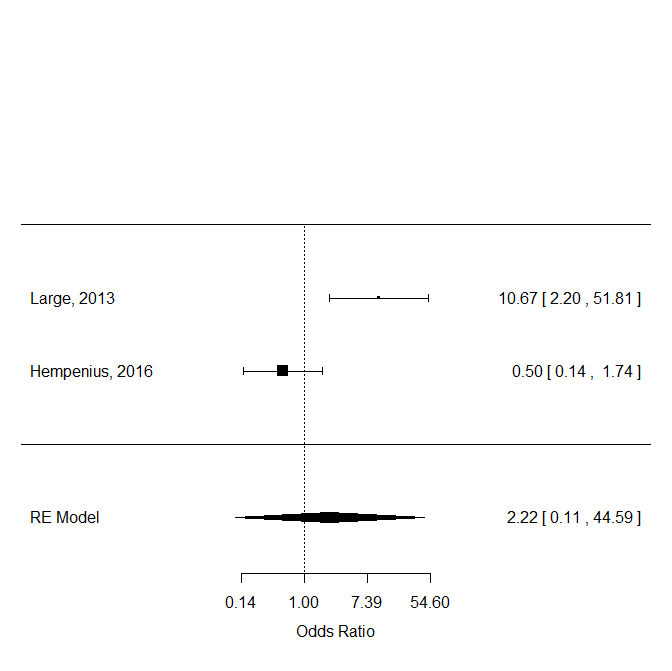


**Figure 41.** Forest plot of the study-level effect measures and the summary effect measure for the odds of readmission to hospital in patients with postoperative delirium (I^2^=88.73%).


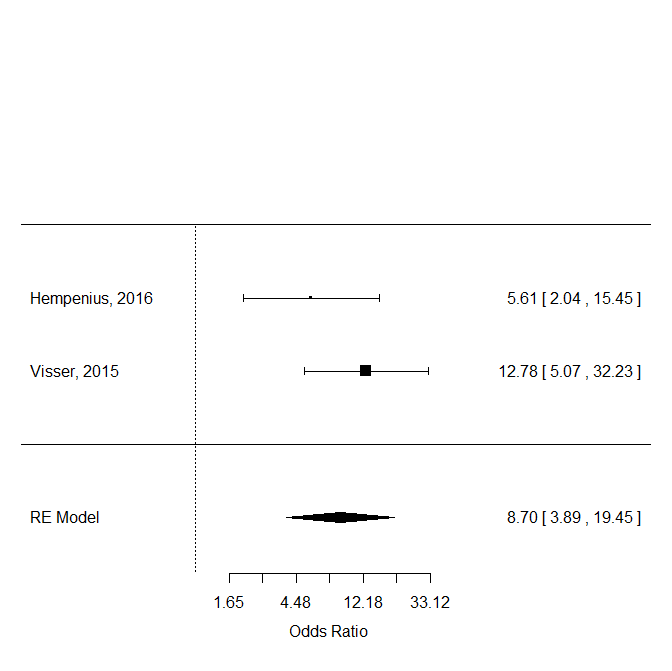


**Figure 42.** Forest plot of the study-level effect measures and the summary effect measure for the odds of non-home discharge in patients with postoperative delirium (I^2^=27.66%).

# eFigure 2. Funnel plots of prospective studies reporting prognostic factors associated with postoperative delirium among older adults undergoing elective surgery


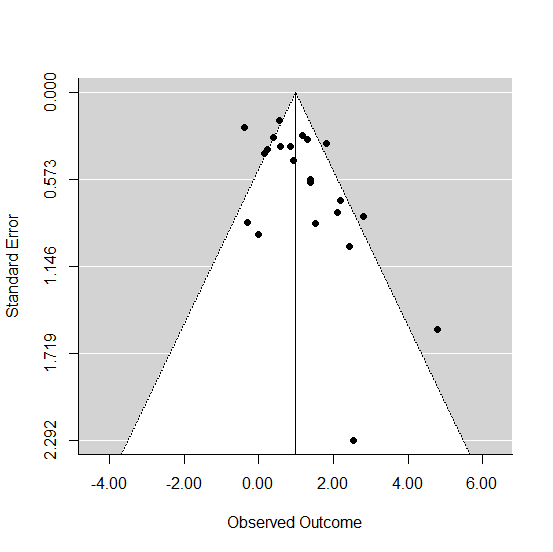


**Figure 1a.** Funnel plot of published studies reporting cognitive impairment as a prognostic factor for postoperative delirium among older adults undergoing elective surgery (Egger’s test p=0.02).


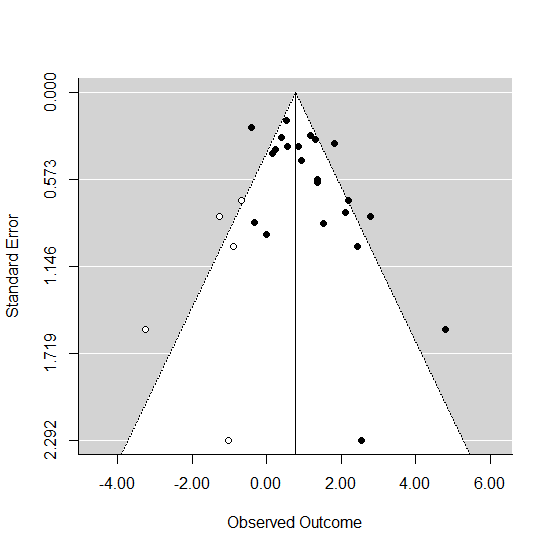


**Figure 1b.** Funnel plot of published studies augmented using the trim-and-fill method reporting cognitive impairment as a prognostic factor for postoperative delirium among older adults undergoing elective surgery (5 filled in studies on the left, OR 2.18, 95% CI 1.48-3.21, p<0.001).


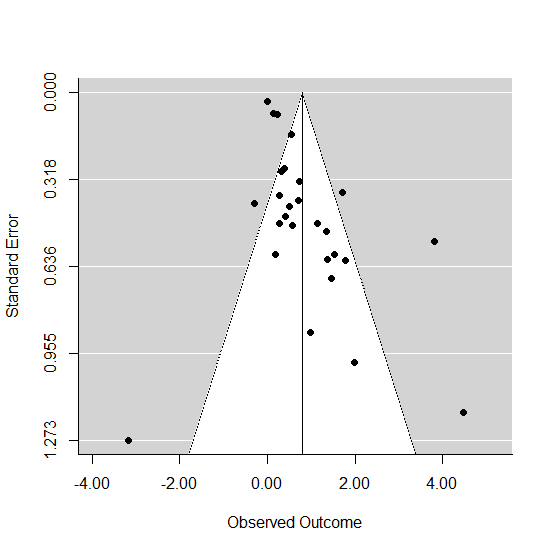


**Figure 2a.** Funnel plot of published studies reporting age as a prognostic factor for postoperative delirium among older adults undergoing elective surgery (Egger’s test p<0.001).

**
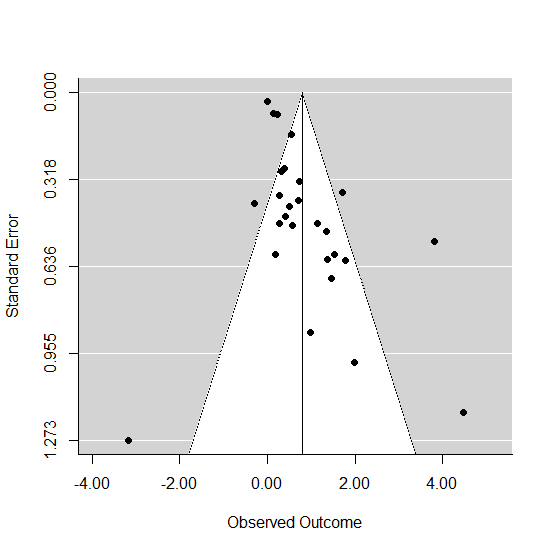
**

**Figure 2b.** Funnel plot of published studies augmented using the trim-and-fill method reporting age as a prognostic factor for postoperative delirium among older adults undergoing elective surgery (0 filled in studies).


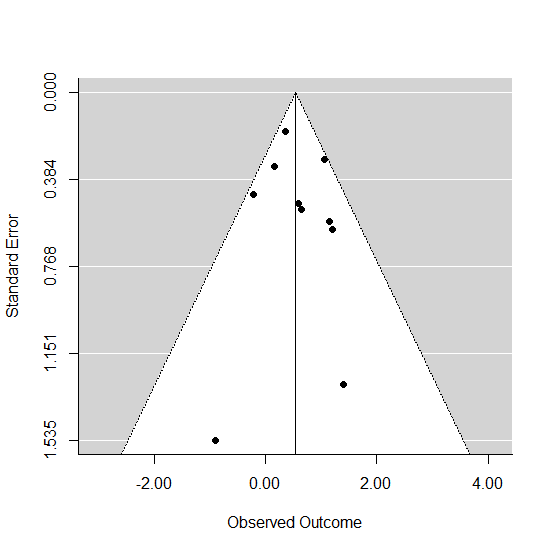


**Figure 3.** Funnel plot of published studies reporting American Society of Anesthesiologists (ASA) status as a prognostic factor for postoperative delirium among older adults undergoing elective surgery (Egger’s test p=0.60).


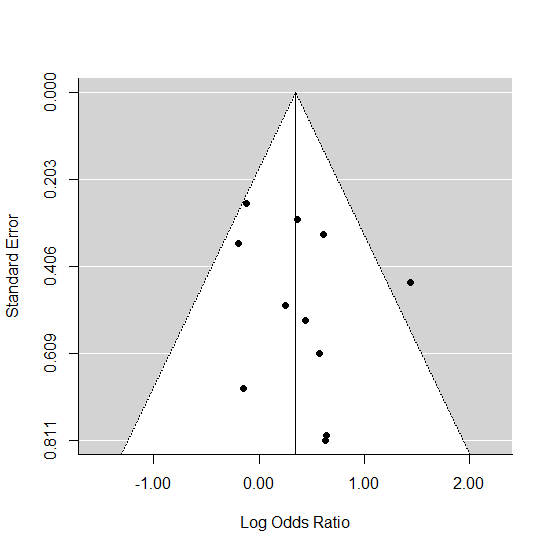


**Figure 4.** Funnel plot of published studies reporting a history of diabetes mellitus as a prognostic factor for postoperative delirium among older adults undergoing elective surgery (Egger’s test p=0.32).


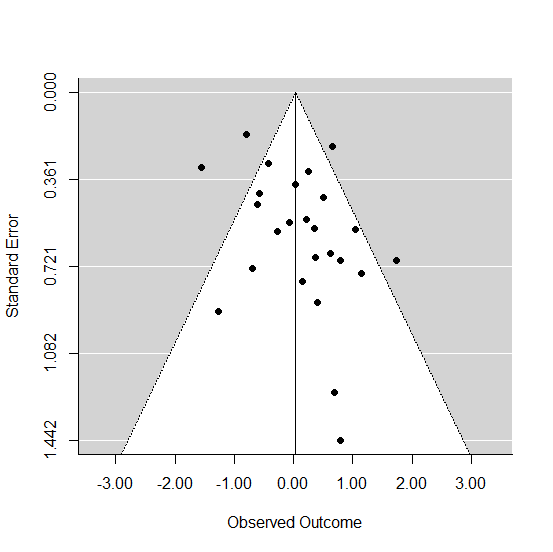


**Figure 5.** Funnel plot of published studies reporting patient sex as a prognostic factor for postoperative delirium among older adults undergoing elective surgery (Egger’s test p=0.06).


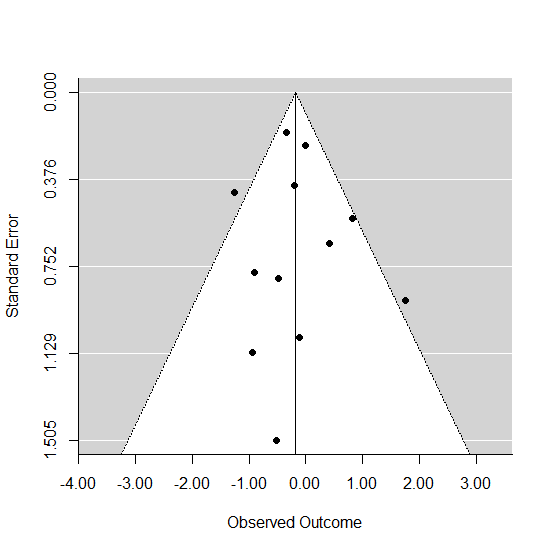


**Figure 6.** Funnel plot of published studies reporting alcohol consumption as a prognostic factor for postoperative delirium among older adults undergoing elective surgery (Egger’s test p=0.70).


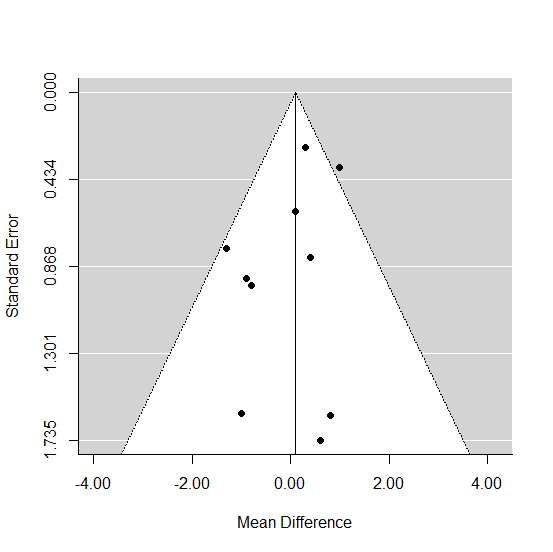


**Figure 7.** Funnel plot of published studies reporting body mass index (BMI) as a prognostic factor for postoperative delirium among older adults undergoing elective surgery (Egger’s test p=0.17).


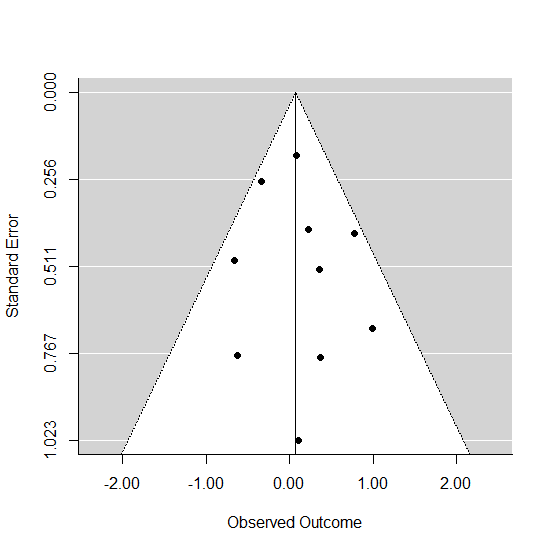


**Figure 8.** Funnel plot of published studies reporting general anesthesia as a prognostic factor for postoperative delirium among older adults undergoing elective surgery (Egger’s test p=0.61).

# References

1. Sanchez-Meca J, Marin-Martinez F, Chacon-Moscoso S. Effect-size indices for dichotomized outcomes in meta-analysis. *Psychol Methods.* 2003;8(4):448-467.

2. Papaioannou A, Fraidakis O, Michaloudis D, Balalis C, Askitopoulou H. The impact of the type of anaesthesia on cognitive status and. *European Journal of Anesthesiology.* 2005;22:492-499.

3. Bakker RC, Osse RJ, Tulen JH, Kappetein AP, Bogers AJ. Preoperative and operative predictors of delirium after cardiac surgery in elderly patients. *Eur J Cardiothorac Surg.* 2012;41(3):544-549.

4. Blakoe M, Greve H. Frailty - preoperative assessment and implications for nursing practice following heart surgery. Paper presented at: EuroHeartCare2015; Dubrovnik, Croatia.

5. Brouquet A, Cudennec T, Benoist S, et al. Impaired mobility, ASA status and administration of tramadol are risk factors for postoperative delirium in patients aged 75 years or more after major abdominal surgery. *Ann Surg.* 2010;251(4):759-765.

6. Cerejeira J, Batista P, Nogueira V, Firmino H, Vaz-Serra A, Mukaetova-Ladinska EB. Low preoperative plasma cholinesterase activity as a risk marker of postoperative delirium in elderly patients. *Age Ageing.* 2011;40(5):621-626.

7. Clement ND, MacDonald D, Howie CR, Biant LC. The outcome of primary total hip and knee arthroplasty in patients aged 80 years or more. *J Bone Joint Surg Br.* 2011;93(9):1265-1270.

8. Dasgupta M, Rolfson DB, Stolee P, Borrie MJ, Speechley M. Frailty is associated with postoperative complications in older adults with medical problems. *Archives of Gerontology and Geriatrics.* 2009;48:78-83.

9. Eriksson M, Samuelsson E, Gustafson Y, Aberg T, Engstrom KG. Delirium after coronary bypass surgery evaluated by the organic brain syndrome protocol. *Scand Cardiovasc J.* 2002;36(4):250-255.

10. Fisher BWF, Gordon. A Simple Model for Predicting Postoperative Delirium in Older Patients Undergoing Elective Orthopedic Surgery. *Journal of the American Geriatrics Society.* 1995;43:175-178.

11. Flink BJ, Rivelli SK, Cox EA, et al. Obstructive Sleep Apnea and Incidence of Postoperative Delirium after Elective Knee Replacement in the Nondemented Elderly. *Anesthesiology.* 2012;116:788-796.

12. Freter SHD, Michael J.; MacLeod, Heather; Morrison, Michelle; MacKnight, Chris; Rockwood, Kenneth. Predicting post-operative delirium in elective orthopaedic patients: the Delirium Elderly At-Risk (DEAR) instrument. *Age and Ageing.* 2005;34:169-171.

13. Fukuse T, Satoda N, Hijiya K, Fujinaga T. Importance of a comprehensive geriatric assessment in prediction of complications following thoracic surgery in elderly patients. *Chest.* 2005;127:886-891.

14. Gani H, Domi R, Kodra N, et al. The incidence of postoperative delirium in elderly patients after urologic surgery. *Med Arch.* 2013;67(1):45-47.

15. Hattori H, Kamiya J, Shimada H, et al. Assessment of the risk of postoperative delirium in elderly patients using E-PASS and the NEECHAM Confusion Scale. *Int J Geriatr Psychiatry.* 2009;24(11):1304-1310.

16. Hempenius L, Slaets JPJ, van Asselt D, de Bock TH, Wiggers T, van Leeuwen BL. Long Term Outcomes of a Geriatric Liaison Intervention in Frail Elderly Cancer Patients. *PloS one.* 2016;11:e0143364.

17. Jankowski CJ, Trenerry MR, Cook DJ, et al. Cognitive and functional predictors and sequelae of postoperative delirium in elderly patients undergoing elective joint arthroplasty. *Anesth Analg.* 2011;112(5):1186-1193.

18. Kim KI, Park KH, Koo KH, Han HS, Kim CH. Comprehensive geriatric assessment can predict postoperative morbidity and mortality in elderly patients undergoing elective surgery. *Arch Gerontol Geriatr.* 2013;56(3):507-512.

19. Koebrugge B, Koek HL, Van Wensen RJA, Dautzenberg PLJ, Bosscha K. Delirium after abdominal surgery at a surgical ward with a high standard of delirium care: Incidence, risk factors and outcomes. *Digestive Surgery.* 2009;26:63-68.

20. Kosar CM, Tabloski PA, Travison TG, et al. Effect of preoperative pain and depressive symptoms on the risk of postoperative delirium: A prospective cohort study. *The Lancet Psychiatry.* 2014;1:431-436.

21. Kristjansson SR, Nesbakken A, Jordh??y MS, et al. Comprehensive geriatric assessment can predict complications in elderly patients after elective surgery for colorectal cancer: A prospective observational cohort study. *Critical Reviews in Oncology/Hematology.* 2010;76:208-217.

22. Kudoh A, Takase H, Takahira Y, Takazawa T. Postoperative confusion increases in elderly long-term benzodiazepine users. *Anesthesia and Analgesia.* 2004;99:1674-1678.

23. Large MC, Reichard C, Williams JT, et al. Incidence, risk factors, and complications of postoperative delirium in elderly patients undergoing radical cystectomy. *Urology.* 2013;81(1):123-128.

24. Leung JM, Sands LP, Lim E, Tsai TL, Kinjo S. Does preoperative risk for delirium moderate the effects of postoperative pain and opiate use on postoperative delirium? *Am J Geriatr Psychiatry.* 2013;21(10):946-956.

25. Min L, Mazzurco L, Gure TR, et al. Longitudinal functional recovery after geriatric cardiac surgery. *Journal of Surgical Research.* 2015;194:25-33.

26. Morimoto Y, Yoshimura M, Utada K, Setoyama K, Matsumoto M, Sakabe T. Prediction of postoperative delirium after abdominal surgery in the elderly. *J Anesth.* 2009;23(1):51-56.

27. Olin K, Eriksdotter-Jonhagen M, Jansson A, Herrington MK, Kristiansson M, Permert J. Postoperative delirium in elderly patients after major abdominal surgery. *Br J Surg.* 2005;92(12):1559-1564.

28. Otomo S, Maekawa K, Goto T, Baba T, Yoshitake A. Pre-existing cerebral infarcts as a risk factor for delirium after coronary artery bypass graft surgery. *Interact Cardiovasc Thorac Surg.* 2013;17(5):799-804.

29. Patti R, Saitta M, Cusumano G, Termine G, Di Vita G. Risk factors for postoperative delirium after colorectal surgery for carcinoma. *Eur J Oncol Nurs.* 2011;15(5):519-523.

30. Priner M, Jourdain M, Bouche G, Merlet-Chicoine I, Chaumier JA, Paccalin M. Usefulness of the short IQCODE for predicting postoperative delirium in elderly patients undergoing hip and knee replacement surgery. *Gerontology.* 2008;54(2):116-119.

31. Raats JW, Steunenberg SL, Crolla RMPH, Wijsman JHH, te Slaa A, van der Laan L. Postoperative delirium in elderly after elective and acute colorectal surgery: A prospective cohort study. *International Journal of Surgery.* 2015;18:216-219.

32. Robinson TN, Wu DS, Pointer LF, Dunn CL, Moss M. Preoperative cognitive dysfunction is related to adverse postoperative outcomes in the elderly. *J Am Coll Surg.* 2012;215(1):12-17.

33. Rogers MP, Liang MH, Daltroy LH, et al. Delirium after elective orthopedic surgery: risk factors and natural history. *Int J Psychiatry Med.* 1989;19(2):109-121.

34. Rolfson DB, McElhaney JE, Rockwood K, et al. Incidence and risk factors for delirium and other adverse outcomes in older adults after coronary artery bypass graft surgery. *Can J Cardiol.* 1999;15(7):771-776.

35. Rudolph JL, Jones RN, Rasmussen LS, Silverstein JH, Inouye SK, Marcantonio ER. Independent vascular and cognitive risk factors for postoperative delirium. *Am J Med.* 2007;120(9):807-813.

36. Santos FS, Velasco IT, Fraguas R, Jr. Risk factors for delirium in the elderly after coronary artery bypass graft surgery. *Int Psychogeriatr.* 2004;16(2):175-193.

37. Sasajima Y, Sasajima T, Azuma N, et al. Factors related to postoperative delirium in patients with lower limb ischaemia: a prospective cohort study. *Eur J Vasc Endovasc Surg.* 2012;44(4):411-415.

38. Suh DH, Kim J-W, Kim HS, Chung HH, Park NH, Song YS. Pre- and intra-operative variables associated with surgical complications in elderly patients with gynecologic cancer: The clinical value of comprehensive geriatric assessment. *Journal of Geriatric Oncology.* 2014;5:315-322.

39. Tai S, Xu L, Zhang L, Fan S, Liang C. Preoperative risk factors of postoperative delirium after transurethral prostatectomy for benign prostatic hyperplasia. *Int J Clin Exp Med.* 2015;8(3):4569-4574.

40. Tognoni P, Simonato A, Robutti N, et al. Preoperative risk factors for postoperative delirium (POD) after urological surgery in the elderly. *Archives of Gerontology and Geriatrics.* 2011;52:e166-e169.

41. Veliz-Reissmuller G, Aguero Torres H, van der Linden J, Lindblom D, Eriksdotter Jonhagen M. Pre-operative mild cognitive dysfunction predicts risk for post-operative delirium after elective cardiac surgery. *Aging Clin Exp Res.* 2007;19(3):172-177.

42. Visser L, Prent A, Van Der Laan MJ, et al. Predicting postoperative delirium after vascular surgical procedures. *Journal of Vascular Surgery.* 2015;62:185-189.

43. Xue P, Wu Z, Wang K, Tu C, Wang X. Incidence and risk factors of postoperative delirium in elderly patients undergoing transurethral resection of prostate: a prospective cohort study. *Neuropsychiatr Dis Treat.* 2016;12:137-142.

44. Higgins J, Green Se. Cochrane Handbook for Systematic Reviews of Interventions Version 5.1.0 [updated March 2011]. The Cochrane Collaboration, 2011. Available from [www.handbook.cochrane.org](http://www.handbook.cochrane.org).
